# Cerebrospinal fluid shunting or dural venous sinus stenting to preserve vision in idiopathic intracranial hypertension (IIH Intervention): protocol for an open-label, multicentre, randomised controlled phase IIb trial

**DOI:** 10.1136/bmjopen-2025-111013

**Published:** 2026-08-03

**Authors:** Georgios Tsermoulas, Susan P Mollan, Victoria Homer, Caroline Kristunas, Phil White, Benjamin R Wakerley, Ahmed K Toma, Fergus Robertson, Gabriele Berman, Fayyaz Ahmed, Denize Atan, Soham Bandyopadhyay, Darren Barton, Thomas Booth, Fion Bremner, Amanda Denton, Jonathan Downer, Richard Edwards, Emma Frew, Jessie Jie Yi Gew, Lisa J Hill, Tom Hughes, Siân Lax, Jason Macdonald, Gemma Maxwell, James McHugh, Pushkar Shah, Esteban Taleti, Rachel Tasker, Shery Thomas, Alexis J Joannides, Alexandra Sinclair

**Affiliations:** 1 Metabolism and Systems Science, University of Birmingham, Birmingham, UK; 2 Department of Neurosurgery, University Hospitals Birmingham NHS Foundation Trust, Birmingham, UK; 3 NIHR Birmingham Biomedical Research Centre, University Hospitals Birmingham NHS Foundation Trust and University of Birmingham, Birmingham, UK; 4 Cancer Research UK Clinical Trials Unit, College of Medicine and Health, University of Birmingham, Birmingham, UK; 5 Newcastle upon Tyne Hospitals NHS Trust, Newcastle University Translational and Clinical Research Institute, Newcastle upon Tyne, UK; 6 Department of Neurology, University Hospitals Birmingham NHS Foundation Trust, Birmingham, UK; 7 Victor Horsley Department of Neurosurgery, NHNN, UCLH NHS Foundation Trust, London, UK; 8 NIHR University College London Hospitals Biomedical Research Centre, UCLH/UCL Joint Research Office, London, UK; 9 Lysholm Department of Neuroradiology, NHNN, London, UK; 10 Birmingham Neuro-Ophthalmology, University Hospitals Birmingham NHS Foundation Trust, Birmingham, UK; 11 Hull Royal Infirmary, Hull University Teaching Hospitals NHS Trust, Hull, UK; 12 Translational Health Sciences, Bristol Medical School, University of Bristol, Bristol, UK; 13 Southampton General Hospital, University Hospital Southampton NHS Foundation Trust, Southampton, UK; 14 King’s College Hospital, King’s College Hospital NHS Foundation Trust, London, UK; 15 School of Biomedical Engineering and Imaging Sciences, King’s College London, London, UK; 16 Department of Neuro-ophthalmology, NHNN, London, UK; 17 IIH UK, Tyne and Wear, UK; 18 Royal Infirmary of Edinburgh, Edinburgh, UK; 19 University Hospitals Bristol and Weston NHS Foundation Trust, Bristol, UK; 20 Department of Applied Health Sciences, University of Birmingham, Birmingham, UK; 21 Cardiff and Vale University Health Board, University Hospital of Wales, Cardiff, UK; 22 Royal Victoria, Newcastle upon Tyne Hospitals NHS Foundation Trust, Newcastle upon Tyne, UK; 23 Sunderland Eye Infirmary, South Tyneside and Sunderland NHS Foundation Trust, Sunderland, UK; 24 Queen Elizabeth University Hospital, NHS Greater Glasgow and Clyde, Glasgow, UK; 25 Leeds General Infirmary, Leeds Teaching Hospitals NHS Trust, Leeds, UK; 26 Queens Medical Centre, Nottingham University Hospitals NHS Trust, Nottingham, UK; 27 Addenbrooke’s Hospital, Cambridge University Hospitals NHS Foundation Trust, Cambridge, UK

**Keywords:** Ophthalmology, Randomized Controlled Trial, Neuroradiology, Quality of Life, HEALTH ECONOMICS

## Abstract

**Introduction:**

Idiopathic intracranial hypertension (IIH) is characterised by raised intracranial pressure (ICP) and typically affects young women with obesity. Patients are at risk of permanent visual loss due to papilloedema. Some require emergency intervention to rapidly reduce papilloedema and preserve vision. The international standard of care for patients with sight-threatening IIH is cerebrospinal fluid (CSF) shunting. However, dural venous sinus stenting (DVSS) is an emerging procedure that is offered at many neuroscience centres internationally as the primary intervention. Currently, there are no randomised controlled trial data supporting the efficacy of any interventional approach for preserving vision in sight-threatening IIH.

**Methods and analysis:**

IIH Intervention is a UK-based two-arm, open-label, multicentre, randomised controlled phase IIb clinical trial with integrated health economic evaluation to compare CSF shunting with DVSS in patients who have confirmed IIH and are at risk of permanent visual loss due to severe papilloedema. The primary outcome is the global thickness of the peripapillary retinal nerve fibre layer (RNFL), an indicator of papilloedema, measured by optical coherence tomography (OCT) over a 6-month period. Secondary outcomes are global thickness of the RNFL over 12 and 24 months, as well as macular ganglion cell layer volume, perimetric mean deviation, headache outcomes, intervention reporting measures (including complications and revisions) and patient-reported outcomes over 6, 12 and 24 months.

**Ethics and dissemination:**

The protocol was approved initially on 12 December 2022 by West Midlands-South Birmingham Research Ethics Committee (ref: 22/WM/0230). Participants will be required to provide written informed consent. The results of this trial will be disseminated through national and international presentations and peer-reviewed publications.

**Trial registration number:**

ISRCTN57142415.

**STRENGTHS AND LIMITATIONS OF THIS STUDY**

- Idiopathic intracranial hypertension (IIH) Intervention is a randomised controlled trial to directly compare the clinical effectiveness of cerebrospinal fluid (CSF) shunting with dural venous sinus stenting (DVSS) providing level 1 evidence for the treatment of sight-threatening papilloedema in people living with IIH.
- IIH Intervention uses optical coherence tomography (OCT) imaging indices as the primary outcome measure in IIH; these more objective and reproducible vision outcomes rely on anatomical measurements to assess treatment response and align with everyday ophthalmology clinical practice.
- Since OCT is a less variable primary outcome, this trial can recruit a smaller sample size than other IIH trials that have depended on other visual outcomes, such as Humphrey visual field, which has been noted to be unreliable in many patients with IIH, particularly those with very high intracranial pressure in the context of sight-threatening IIH.
- IIH Intervention includes a health economic evaluation comparing the cost-effectiveness of CSF shunting versus DVSS, providing evidence to inform policy decisions.
- Optic nerve atrophy and a reduction in papilloedema can both cause a pseudo-reduction in the global thickness of the peripapillary retinal nerve fibre layer, the primary outcome of this trial.
- Although the macular ganglion cell layer is being monitored and reported to distinguish between these effects, this may affect the interpretation of the trial.

## Introduction

 Idiopathic intracranial hypertension (IIH) is a condition characterised by elevated intracranial pressure (ICP) of unknown aetiology.^1^ IIH prevalence is higher among women of childbearing age with raised body mass index and the incidence is rising in line with the global obesity epidemic.^2 3^ People with IIH are increasingly recognised as having a systemic metabolic disorder, suggesting the condition may not be truly idiopathic.^1^ They exhibit a distinct androgen excess profile^4^ and markers of metabolic dysfunction, with an elevated risk of cardiovascular disease.^1 3^ Morbidity in IIH is high due to the risk of visual loss (up to 25%) from papilloedema and chronic disabling headaches that are refractory to current treatment.^5–7^ There are other disease features that are increasingly being recognised such as depression and anxiety,^8^ cognitive change^9^ and reproductive health morbidity.^10 11^ Many patients with IIH are managed conservatively (93%) with weight loss and pharmacotherapies.^2 12–14^ However, a proportion (<10%) of patients develop fulminant sight-threatening papilloedema and require urgent surgical intervention to rapidly reduce the optic nerve swelling and prevent severe sight loss.^2 15 16^

Internationally, the most commonly used surgical intervention for patients with IIH at risk of permanent visual loss from papilloedema is cerebrospinal fluid (CSF) shunting, typically with a ventriculoperitoneal (VP) or lumboperitoneal (LP) shunt.^12 15 17^ Several open-label trials and case series have shown that CSF shunting is beneficial in patients with IIH at risk of permanent visual loss. However, reported rates of shunt complications and revisions vary widely (11%–71%), and the quality of evidence is low due to the absence of randomised studies and the use of heterogeneous, not directly comparable outcomes, such as complication, revision or failure rates, measured over differing follow-up periods. However, most complications are reported within the first year.^18–30^

An alternative surgical intervention for sight-threatening IIH is dural venous sinus stenting (DVSS), a procedure carried out by neurointerventionists using stents to mechanically expand stenoses within the dural venous sinuses.^31 32^ Recent systematic reviews and meta-analyses from open-label studies have indicated that DVSS may reduce the signs and symptoms of IIH, but the efficacy of DVSS to preserve vision function remains unclear.^33 34^ A review of multiple recent studies involving 825 patients reported improvements in papilloedema in 87% of patients, headache in 72% and visual acuity in 65%; however, all were retrospective and used different methodologies and inclusion criteria.^35^ Data on revision rates are encouraging, open-label data from 41 patients at a single centre reported stent revision rates of 14% within the first year.^36^ A more recent review analysing 1626 patients from 45 retrospective and four prospective studies, together with 18 case reports, reported improvements in ICP, visual symptoms and headaches poststent, and highlighted the need for a prospective head-to-head trial to better understand the performance of stenting compared with other interventions for IIH.^37^ Although currently not recommended by the IIH consensus guidelines for the acute management of sight-threatening IIH,^12^ DVSS is being offered at many large neuroscience centres internationally as a primary intervention.

There are no randomised controlled trials in IIH comparing CSF shunting with DVSS and there remains clinical equipoise about which intervention is best in sight-threatening IIH. Determining the best procedure to preserve vision in IIH was also identified as an unmet need and a top research priority from the patients in a James Lind research priority setting partnership.^38^ Therefore, the aim of this trial is to evaluate and compare the clinical and cost-effectiveness of these two interventions.

## Methods and analysis

### Patient and public involvement

The Patient and Public Advisory Group (PPAG) is led by IIH UK, a registered charity in England and Wales (no 1143522); they co-developed the IIH Intervention trial protocol design and methodology. Specifically, the PPAG reviewed and refined the protocol providing input into the acceptability of the visit schedule, patient-facing materials such as the information sheets and patient video as well as input into trial outcome measures relevant to patients and patient-reported outcomes. As members of the Trial Steering Committee (TSC), the PPAG will assess study conduct, will be involved in the recruitment to the trial by educating patients with IIH about research and the importance of the trial question and will support dissemination of the study results via patient conferences, websites and social media run by IIH patient charities. Patient education meetings will be held in collaboration with IIH UK to discuss the current equipoise in the evidence base and to highlight the importance of the trial, with the aim of supporting patient acceptability and to enhance recruitment and participant retention in the trial.

### Trial design

**Figure 1:**
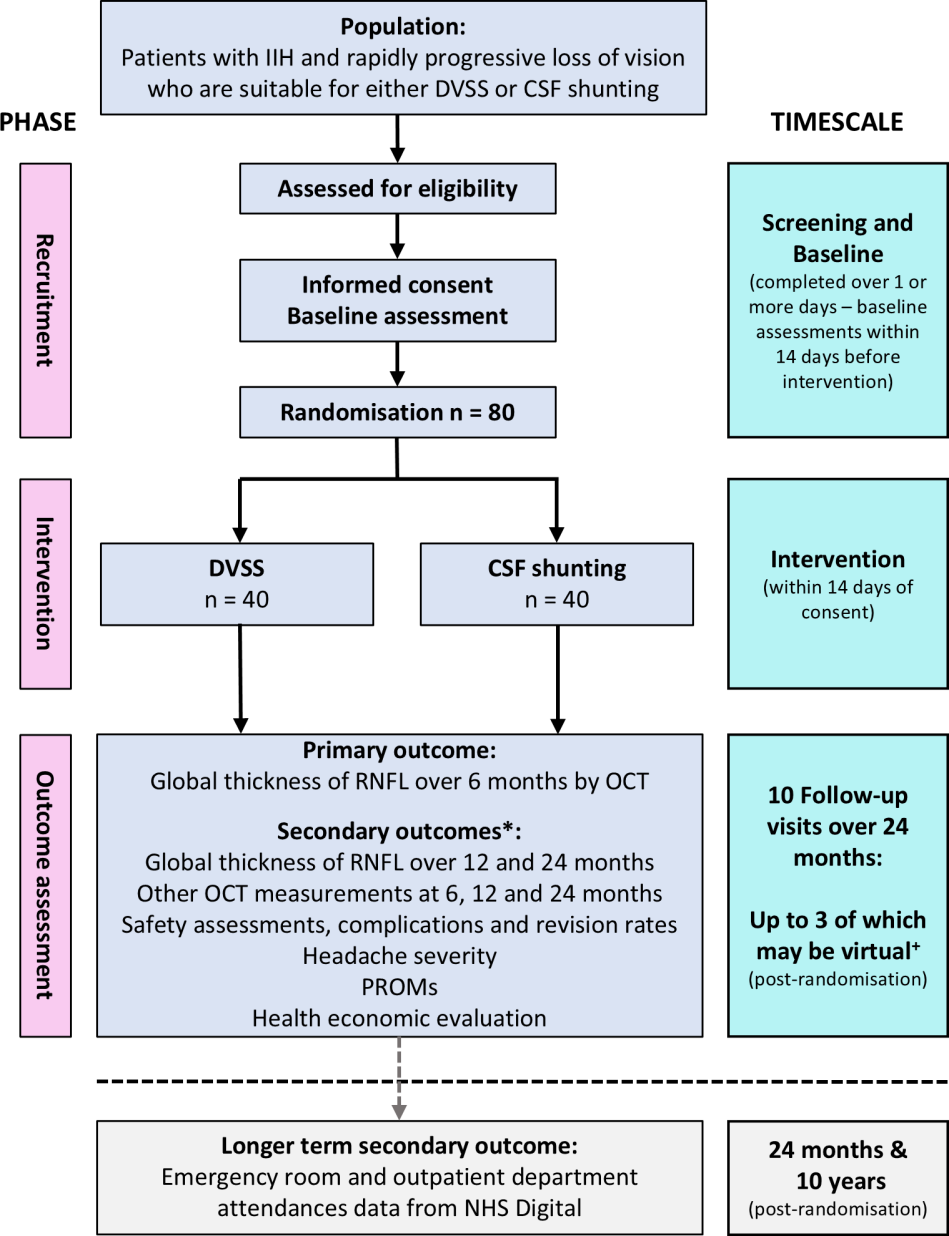
IIH Intervention trial diagram. An overview of the IIH Intervention trial. *For a full list of secondary outcomes, please refer to the text and online supplemental appendix 5. + Sites will be allocated two early visits that can be conducted over the phone or as a virtual appointment. Possible virtual visits are at months 2, 3 or 4. See ‘Discussion’ section for details. In addition, the 18-month visit may be conducted over the phone or as a virtual appointment. CSF, cerebrospinal fluid; DVSS, dural venous sinus stenting; IIH, idiopathic intracranial hypertension; NHS, National Health Service; OCT, optical coherence tomography; PROMs, patient-reported outcome measures; RNFL, retinal nerve fibre layer.

IIH Intervention is a two-arm, open-label, multicentre, randomised controlled phase IIb clinical trial with an integrated health economic evaluation. All recruitment centres are National Health Service (NHS) hospitals in the UK. The trial will compare CSF shunting with DVSS, the two most common interventions performed in the UK for patients who have confirmed IIH and are at risk of permanent visual loss due to papilloedema (figure 1).

During the 24-month follow-up period, participants will have regular contact with the participating clinical and research teams. These contacts will be in-person (defined clinic visits) and virtual, according to the participant pathway (online supplemental appendix 1). In addition, linked-anonymised routinely collected patient health data will be obtained (where possible) from NHS Digital^39^ from 2 years and up to 10 years post-intervention (even in those participants who discontinue from trial research follow-up as long as they do not specifically request full trial withdrawal).

All items from the WHO Trial Registration Data Set are included in online supplemental appendix 2.

The full protocol can be requested from the Trial’s Office (iihintervention@trials.bham.ac.uk).

### Patient selection

This trial is recruiting patients with IIH who have sight-threatening papilloedema at risk of permanent visual loss, a condition for which there is currently no evidence-based or historical definition. The definition below is used in this trial:

- In a patient with confirmed IIH, visual loss, secondary to papilloedema that cannot be explained by other ocular or central nervous system (CNS) pathology in which there is increasing papilloedema with Frisén grade ≥3 and increasing papilloedema demonstrated on OCT imaging, in conjunction with loss (or potential for loss) of perimetric mean deviation (PMD).
- Where there is no preceding background information to establish a change in vision, we define the risk of permanent visual loss as papilloedema Frisén grade ≥3, in conjunction with loss of PMD.
- As visual field loss in IIH typically develops over time, acute cases may initially present with minimal PMD loss. In such situations, intervention may be required because of the severity of papilloedema and raised ICP to prevent the development of a visual field deficit, which can be permanent.

Eligibility criteria for participants are listed in box 1.

Box 1 **Key patient eligibility criteria for the idiopathic intracranial hypertension (IIH) Intervention trial**

**Inclusion criteria**

- Diagnosis of IIH.^1 2^
- Presence of papilloedema (Frisén grade ≥3) in at least one eye.
- Age 18 years to <64 years at the time of consent.
- Patients must be suitable for and willing to proceed with both cerebrospinal fluid (CSF) shunting (ventriculoperitoneal or lumboperitoneal shunts only) and dural venous sinus stenting (DVSS).
- Able to provide written informed consent.

**Exclusion criteria**

- Presence of current venous sinus thrombosis on diagnostic brain imaging by either MRI, magnetic resonance venography or computed tomography venography.
- Previous surgery for IIH, including optic nerve sheath fenestration, CSF shunting procedures, subtemporal decompression and DVSS.
- Previous bariatric surgery within the last 3 months.
- Patients with an ophthalmic history, except refraction error, affecting the eligible eyes (study eyes) that could affect the vision.
- Patient is, at the time of signing the informed consent, a user of recreational or illicit drugs (including marijuana) or has had a recent history (within the last year) of drug or alcohol abuse or dependence, in the opinion of the investigator.
- History of any clinically significant disease or disorder which, in the opinion of the investigator, may either put the subject at risk because of participation in the study, or influence the results or the subject’s ability to participate in the study.
- Have participated in any other interventional study within 30 days prior to the screening visit (of note participation in the IIH Life database or other observational studies will not prevent enrolment to this study).
- Previous randomisation for treatment in the present study.
- Pregnant.
- Absolute or serious contraindication to the standard antithrombotic regimen during the peri-stenting and post-stenting periods.
- History of significant documented iodine-based contrast allergy.
- History of documented allergy to nitinol or nickel.
- Absolute or serious contraindication for general anaesthesia.
- Previous diagnosis of a hypercoagulable state (factor V Leiden, protein C or S deficiency, anticardiolipin antibodies, lupus anticoagulant, B2-glycoprotein-1 antibodies or hyperhomocysteinaemia).
- Currently requiring full anticoagulation for other medical reasons, such as atrial fibrillation, artificial valves, deep vein thrombosis or pulmonary embolism.
- Documented prior non-traumatic intracranial haemorrhage.
- History of deep vein thrombosis or pulmonary embolism (within the last 24 months).
- History of severe carotid atherosclerotic disease.
- History of heart failure, dilated cardiomyopathy or congenital heart disease that are assessed as at high thrombotic risk.
- Presence of intracranial vascular malformation considered clinically symptomatic (current or within 24 months).
- Anatomical anomaly of the venous sinus, which would prevent safe catheterisation and stenting (eg, multichannel sinus).

### Consent

Eligible patients will be invited to participate by a member of the research team at each site before any trial-related procedures. Exemplar patient information sheets and consent forms are included in online supplemental appendices 3 and 4, respectively. A patient information video is also used to support the consent process. Within this process, consent is optional for additional blood samples to be taken.

### Randomisation

Participants are randomised by the research team at each site. The individuals at each site who enrol and randomise participants do not have access to the randomised sequence. Randomisation is on a 1:1 basis produced by a computer-generated randomisation via the trial’s online electronic remote data capture (eRDC) system operated by the Cancer Research UK Cancer Clinical Trials Unit (CRCTU), University of Birmingham. Treatment allocation is stratified by the following criteria:

1. Presence of or intention to insert lumbar drain prior to the protocol-defined intervention.
2. Duration of IIH diagnosis (diagnosis ≤28 days from randomisation or diagnosis >28 days from randomisation).
3. The degree of papilloedema defined by the Frisén grade being <4 or ≥4 in the most affected study eye.

To reduce the risk of any possibility of the treatment allocation becoming predictable, the randomisation algorithm includes a random factor, whereby 80% of randomisations will be ascertained using the minimisation allocation, with the remaining 20% being true randomisation. This approach preserves allocation concealment while maintaining group balance.

### Interventions

Standard operating procedures (SOP) have been developed for both CSF shunting and DVSS for guidance only, as both interventions, including peri-operative management, will be carried out as part of standard clinical care. The treating clinicians will perform the interventions in accordance with local practice and the details of the procedures (procedural technique, equipment, stent type, shunt components) will be collected for exploratory analysis of secondary outcomes. Adherence to the randomised intervention will be recorded, along with technical success and any failed procedures.

### Cerebrospinal fluid shunting

Insertion of a CSF shunt is one of the most common neurosurgical procedures and will be carried out by a neurosurgeon according to local practice. Either a VP or a LP shunt can be offered based on the surgeon’s preference and on individual participant factors, such as size of the ventricles. Other types of shunts, including ventriculoatrial and ventriculopleural shunts, are only rarely used as primary treatment and are, therefore, not included in the trial to avoid heterogeneity.^12^ Any type of shunt valve or other component can be used, based on local practice. The peri-operative radiological imaging is also performed as part of routine care.

### Dural venous sinus stenting

Before the opening of the trial, DVSS was not performed in all participating sites and, therefore, the trial’s DVSS accreditation committee reviewed the experience and expertise of the interventional neuro-radiology service at each site to ensure competency at performing the procedure (see ‘Discussion’ section). Like CSF shunting, DVSS will be carried out according to local practice with the consensus aiming to treat the dominant stenosis (as judged by the local team) in a transverse/sigmoid venous sinus segment. If during the angiography, a targetable stenosis is not identified, the procedure should be abandoned as per standard practice. A threshold pressure gradient across the stenosis is not universally accepted and no gradient is defined in the protocol. Furthermore, under general anaesthesia, pressure gradients can deviate considerably from baseline. Of note, none of the currently available stents in the market are licensed for use in the dural venous sinuses. The peri-operative radiological imaging and use of antiplatelet agents to prevent stent thrombosis will follow local practice.

### Intervention failure

An intervention failure usually refers to a mechanical complication or infection for a CSF shunt and to thrombosis or re-stenosis for a stent. Intervention failure is categorised as:

- Technical failure without recurrence of papilloedema.
- Failure with ongoing or recurrent papilloedema.
- Failure to manage ongoing headache (in the absence of ocular disease progression).
- Failure with neurological complications or local/systemic complication (eg. infection).
- Other.

When failure occurs, rescue therapy may be initiated based on the clinical judgement of the treating physicians. The choice of rescue therapy will be documented and reported and may include an ICP-lowering medication or a further surgical intervention, such as a revision procedure or an alternative surgical intervention. If an alternative surgical procedure is conducted, this will be recorded.

### Other procedures and medications

Migraine preventative medication and the use of acute headache analgesics are permitted. Bariatric surgery and optic nerve sheath fenestration are not; however, if they occur as a protocol deviation, they will be accounted for in the statistical analysis plan (SAP; available on request to the Trial’s Office (iihintervention@trials.bham.ac.uk)). Weight loss medications, if used, will be recorded and the amount of weight lost will also be accounted for in the SAP.

### Trial outcomes

**Figure 2:**
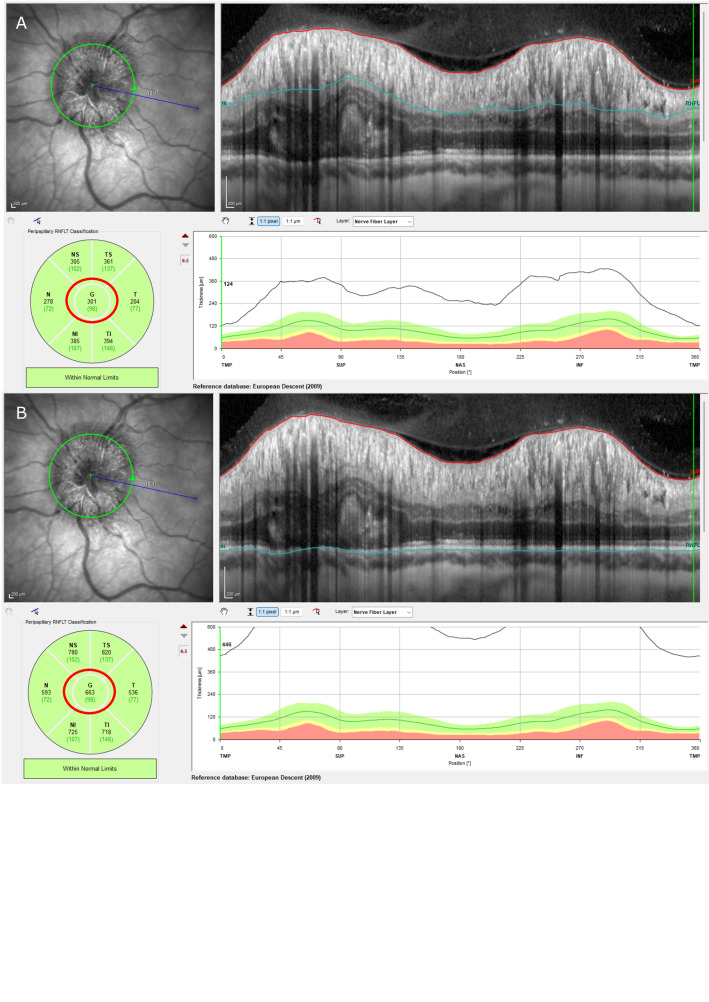
Primary outcome measured by optical coherence tomography (OCT). (**A**) Peripapillary retinal fibre layer (RNFL) OCT scan, Spectralis (Heidelberg Engineering), showing the global RNFL thickness. Manual resegmentation is performed as required. Red circle: global RNFL 301 μm. (**B**) Retinal nerve fibre layer scan, Spectralis OCT scan (Heidelberg Engineering), showing the total retinal thickness (TRT), which is measured from internal limiting membrane (red) to Bruch’s membrane (blue). Manual resegmentation is performed as required. Red circle: global TRT 663 μm. G, Global; INF, inferior; N, nasal; NAS, nasal; NI, nasal inferior; NS, nasal superior; SUP, Superior; T, Temporal; TI, temporal inferior; TMP, Temporal; TS, temporal superior.

The primary outcome is global thickness of the peripapillary retinal nerve fibre layer (RNFL), as measured using OCT Spectralis (Heidelberg Engineering), over 6 months (figure 2).

**Figure 3:**
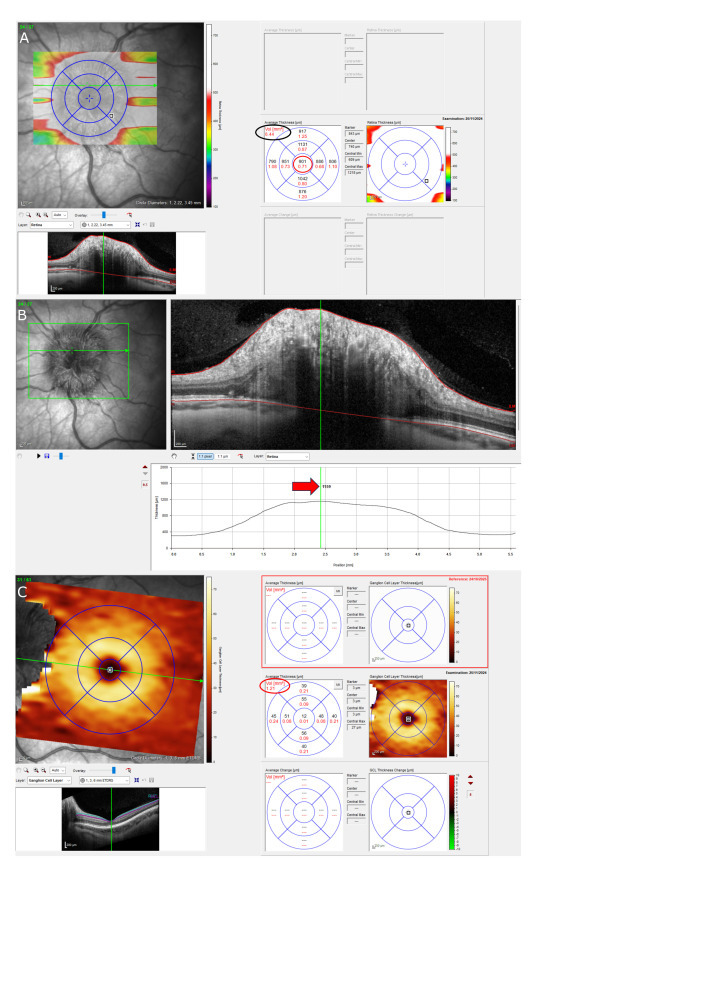
Secondary outcomes measured by optical coherence tomography (OCT). (**A**) Optic nerve head volume scan, Spectralis OCT scan (Heidelberg Engineering), showing the optic nerve (disc) global volume and optic nerve head (disc) central thickness. Manual resegmentation is performed as required. Black oval: optic nerve global volume 8.44 mm^3^. Red circle: optic nerve head central thickness 901 μm. (**B**) Optic nerve head volume scan, Spectralis OCT (Heidelberg Engineering), showing the optic nerve head (disc) maximum height. This parameter requires manual identification of the B-scan with highest profile. Red arrow: maximum height 1159 μm. (**C**) Posterior pole scan, Spectralis OCT (Heidelberg Engineering), showing the macular ganglion cell layer (GCL) volume. Red circle: macular GCL volume 1.21 mm^3^.

Secondary outcome measures include global thickness of the RNFL by OCT over 12 and 24 months. Other measures will be evaluated over 6, 12 and 24 months and include alternative OCT optic nerve head measures of papilloedema (total retinal thickness, disc global volume, disc central thickness and disc maximum height) and the OCT measure indicating optic nerve atrophy (macular ganglion cell layer volume) (figure 3).^40^ A trained and certified technician or neuro-ophthalmologist will evaluate each OCT image to confirm scan quality and ensure accurate segmentation before reporting. Other vision outcome measures are visual acuity, measured by logarithm of the minimum angle of resolution units and Humphrey visual field (HVF) PMD. To ensure standardised, high-quality data, the Visual Reading Centre required all sites to undergo training according to a predefined SOP. Site technicians were accredited prior to site initiation. A detailed list of the other secondary outcomes including safety assessments, intervention reporting measures (including complications and revisions), headache outcomes (including monthly headache days (MHD), headache severity (Numerical Rating Scale (NRS) 0–10) and monthly use of acute headache analgesics), patient-reported outcome measures and a health economic evaluation, as well as the exploratory outcomes are included in online supplemental appendix 5.

### Statistical analysis plan

#### Sample size

There is no established threshold for the minimum clinically important change in RNFL thickness that would define a successful outcome of intervention in patients with IIH.^41^ We analysed optical coherence tomography (OCT) RNFL global thickness in a comparable IIH population undergoing CSF shunting at the Queen Elizabeth Hospital, Birmingham, UK between 2019 and 2021. The cross-sectional SD was 128 µm (OCT RNFL global thickness pre-intervention: 364 µm) and data from logistical regression models illustrated values of 253.9 µm at 2 weeks, 202.6 µm at 1 month, 90.0 µm at 3 months and 87.2 µm at 6 months post-intervention.^42^

Using a conservative correlation between within-subject serial measurements (rho, ρ) of 0.5 and a minimally clinically important difference in global RNFL of 70 µm, it was estimated that this trial could show a potential benefit of an intervention with a sample size of 66 patients, each providing a complete dataset of all indices across all scheduled follow-up visits.

As this trial is longitudinal, we expect that some participants will withdraw from the study or fail to complete a visit, leading to <100% data collection for the primary outcome. In the two arms of a historical IIH trial,^43 44^ the proportion of missing data was 6.5% and 6.7%. Therefore, we expect missing data in approximately 6.6% of values; 95% CIs using Wilson’s method are 2.6% to 15.7%. For our sample size estimation, we use the upper limit of this and increase the number of complete case series by 16%.

We plan to use hierarchical models to longitudinally analyse OCT. Therefore, the methods of Diggle *et al*^45^ are used to derive the sample size. With assessments at trial entry (days −14 to 0), weeks 1 and 2 and months 1, 2, 3, 4 and 6, and in line with the values given above, a minimum of 40 participants per arm are required; at least 80 participants in total.

As noted in figure 1, online supplemental appendix 1 and in the ‘Discussion’ section, sites have the option to perform two of three early visits (at months 2, 3 and 4) virtually, whereby ophthalmology data would not be collected. If two visits are virtual then the power estimate for the trial’s primary outcome is 84%. However, we anticipate our power to be higher than this (either close to or >90%) as:

- Participants will likely contribute data at one of these visits and the allocation process provides a level of exchangeability between participants across different time points.
- The sample size calculation assumes only one outcome measure per participant per time point. Analysis at the eye level (whereby all study-eyes are nested within participants; see the ‘Outcome analyses’ section for more detail) will increase the effective sample size and thereby the power.^46^
- The inflation for missing data assumes that whole participant series are removed; however, we expect visits to either be missing at random or outward censoring (where withdrawal occurs).

Results of a simulation study assessing these points are included in the trial’s SAP; available on request to the Trial’s Office (iihintervention@trials.bham.ac.uk).

#### Outcome analyses

Detailed outcome measure analyses can be found in the predefined SAP; available on request to the Trial’s Office (iihintervention@trials.bham.ac.uk). Briefly, RNFL will be measured immediately prior to treatment commencement (baseline measurement) and then at each of the onsite visits over the 24 months post-intervention (including unscheduled visits for suspected device failure). These measurements will be analysed using hierarchical repeated measures models, only the first 6 months of which will be used in the analysis of the primary outcome. The analysis will include all randomised patients, grouped according to the intervention to which they were assigned.

A random-effects structure will be used to handle repeated measures nested within one eye through time, with each eye nested within a participant. Analysing data from each eye, as opposed to the worst eye, will increase statistical efficiency.^46^ However, there are analysis inclusion criteria at the eye level, due to the possibility that some patients will have only one eye that meets the eligibility criteria (the study eye). In these instances, only measures taken from the study eye will be used in the analysis (eg, where there are non-IIH causes of disease within an eye or an IIH eye that does not meet the eligibility criteria; any measurements taken will not be analysed). Moreover, where data from each eye are analysed, it is imperative to handle the high correlation between eyes within individuals.^46^

Hierarchical models will be used to model both eyes, which are assumed to be nested within participants. These models will therefore have a fixed intercept and fixed effects for the progression of RNFL thickness through time (both with and without a treatment interaction), adjustments for covariates (including but not limited to stratification variables (see ‘Randomisation’ section)) and random effects for the effect of participant on the intercept (participant random intercepts) and the effect of eye within participant on the intercept and slope (eye within participant random intercept and slope). If this model does not fit the observed data satisfactorily, alternative models and explanatory variables (such as age, sex and treating centre) will be considered. The presence of a treatment effect will also be tested by a likelihood ratio test against a nested model with no treatment variable and otherwise identical model specification.

Other visual outcomes will be analysed in a similar way as they can be assumed to be continuous. For the analysis of global RNFL thickness, we acknowledge that loss of the ganglion cell layer (GCL) may cause a pseudo-reduction in RNFL. Therefore, in addition to the primary estimand detailed above, we aim to differentiate any reduction in RNFL owing to an improvement in disease following intervention from any reduction in RNFL owing to the loss of GCL. Therefore, we propose a supplementary estimand, which will be analogous to the analysis detailed above, with the key difference being that GCL will also be adjusted for through the joint modelling of both. This analysis will have the added benefit of allowing the ascertainment of whether the rate of GCL death is comparable between arms.

The major attributable complication rate between the DVSS and CSF shunting arms will be compared using a two-sample test for a difference in proportions.

Headache outcomes, including MHD will be analysed using multilevel negative binomial models with constant overdispersion parameter. These models will have fixed terms for the mean baseline value, the assessment times and treatment arm, with random intercepts to reflect the heterogeneity in baseline values and the serial correlation in repeated measures. Akin to the primary outcome, the presence of a treatment effect will again be tested by a likelihood ratio test against a nested model with no treatment variable and otherwise identical model specification.

All other outcomes, their intercurrent events of interest and estimands are given in the SAP.

Supplementary subgroup analyses may be performed by shunt type (LP vs VP CSF shunt) against DVSS, as appropriate.

There are no formal interim analyses with associated decision criteria. Instead, the independent data monitoring committee (DMC) will meet at least annually, and be presented with interim analyses, including safety and primary outcome data.^41^

#### Economic evaluation

To assess the cost-effectiveness of DVSS compared with CSF shunting, a within-trial economic evaluation will be undertaken and will include both a cost-effectiveness analysis and a cost-utility analysis at 12 months. If the economic evaluation at 12 months reveals that DVSS is not dominated by CSF shunting (ie, it is not both more costly and less effective) then a decision-analytic model will be developed to enable an economic analysis over a longer time horizon. The model will be populated using trial data collected at 24 months, as well as data from other sources to extrapolate results over a lifetime horizon. The economic evaluation will be undertaken from a UK health system perspective.

For the cost-utility analysis, quality-adjusted life years (QALYs) will be measured using the EQ-5D-5L collected at baseline, 1 and 2 weeks immediately following the intervention and then at 1, 4, 6 and 12 months and at any unscheduled visit for revision/device failure.

Utility weights will be obtained by using the EQ-5D index tariff. Mean differences in EQ-5D between the trial arms will be estimated and presented with statistical tests of significance. Any imbalance in baseline utility will be adjusted. The within-trial cost-effectiveness analysis will use ‘major complications avoided’ as the outcome measure, assessed using the Clavien-Dindo grade III–V criteria for classification.^47^

#### Blood samples

Optional blood samples (serum and plasma) at baseline and 12 months will be collected, processed, stored on site and transported in batches to the University of Birmingham for long-term storage in accordance with the Human Tissue Authority guidelines and NHS trust policies. The samples will be analysed to identify potential biomarkers in future research, which will be subject to separate ethics approval.

#### Adverse events and medical device deficiencies

Adverse events (AEs) will be reported in accordance with Clavien-Dindo classification^47^ and/or National Cancer Institute Common Terminology Criteria for Adverse Events, V.5.0.^48^ Definitions of different types of AEs and medical device deficiencies are listed in online supplemental appendix 6. The reporting period for AEs will be between consent until 360 days after initial trial intervention. In addition, any medical device deficiencies will be recorded up to 24 months post-trial intervention specifically related to the medical device allocated at randomisation. The investigator should assess the seriousness and causality (relatedness) of all AEs experienced by the participant (this should be documented in the source data) with reference to the protocol. This will include abnormal laboratory findings, which are reported as clinically significant. All AEs will be reported using the applicable electronic case report form (eCRF).

The investigator will exercise their clinical judgement in deciding whether an abnormal laboratory finding or other abnormal assessment is relevant. However, if in the opinion of the investigator, the frequency or severity of the event is greater than would be expected then it must be reported.

Participants receiving CSF shunting or DVSS as the primary trial intervention may require admission to hospital for appropriate medical intervention following development of some of the more severe known side effects of the intervention (online supplemental appendix 7). The following are regarded as expected serious AEs (SAEs) for the purpose of this study:

- SAEs relating to CSF shunting unless the condition is life threatening or proves fatal.
- SAEs relating to DVSS unless the condition is life threatening or proves fatal.

Any death occurring during the reporting period, whether considered device-related or not, must be reported as an SAE within 24 hours of the local investigator becoming aware of the event. If a death is considered potentially related to either the CSF shunt or stent, then the cause of death will be investigated and reviewed by the trial management group (TMG), DMC and clinical team caring for the participant. Where the death is considered as potentially related to the DVSS intervention, entry of patients into the trial will be temporarily suspended until these investigations are complete.

All SAEs that are at least possibly related to the trial intervention and are still present at the end of the trial will be followed at least until the final outcome is determined. Even if it implies that the follow-up continues after the participant leaves the trial and, where appropriate, until the end of the planned period of follow-up.

#### Data management

An eRDC system is used to capture all trial-related data via eCRFs. All trial data are processed and stored in a secure database, access to which is granted by the Trial’s Office (iihintervention@trials.bham.ac.uk). Paper CRFs must be completed, signed and dated and returned to the Trial’s Office by the investigator or an authorised member of the research team. Data reported on each CRF should be consistent with the source data or the discrepancies should be explained. If information is not known, this must be clearly indicated on the CRF. All missing and ambiguous data will be queried. All sections are to be completed.

The linked-anonymised routinely collected patient health data collected as two separate batches at defined time points will be stored and used in accordance with NHS Digital requirements as per a Data Sharing Agreement.

All trial records will be archived and securely retained for at least 15 years. No documents will be destroyed without prior approval from the sponsor, via the central IIH Intervention Trial’s Office. On-site monitoring will be conducted as required following a risk assessment and as documented in the Quality Management Plan. Any monitoring activities will be reported to the central Trial’s Office and any issues noted will be followed up to resolution. IIH Intervention will also be centrally monitored, which may trigger additional on-site monitoring. Further information regarding data management is provided in the study protocol.

The trials unit will hold the final trial dataset and will be responsible for the controlled sharing of anonymised clinical trial data with the wider research community to maximise potential patient benefit while protecting the privacy and confidentiality of trial participants. Data anonymised in compliance with the Information Commissioner’s Office requirements, using a procedure based on guidelines from the Medical Research Council Methodology Hubs, will be available for sharing with researchers outside of the trials team within 12 months of the primary publication.

#### Trial organisation structure

The University of Birmingham will act as a single sponsor for this multicentre study: Research Ethics, Governance and Integrity, Research Strategy and Services Division, Research Park, Birmingham, B15 2TT (email: researchgovernance@contacts.bham.ac.uk). The trial is being conducted under the auspices of the CRCTU, University of Birmingham according to their local procedures. The TMG is responsible for the day-to-day running and management of the trial. Members of the TMG include the chief investigator, co-investigators and trial team including trial statisticians. The TMG meet at least quarterly.

The TSC is set up with an independent chairperson to oversee the study. Membership is composed of selected TMG members, independent clinicians, a statistician and one patient advocate. The TSC supervises the conduct of the study, monitors progress, including recruitment, data completeness, losses to follow-up and protocol deviations, and assumes responsibility for the trial oversight on behalf of the sponsor. The TSC meets at least yearly.

An independent DMC consists of an independent chair, three additional independent clinicians and an independent statistician.^41^ It advises on whether the accumulated data from the trial, together with results from other relevant research, justifies the continuing recruitment of further patients. The DMC operates in accordance with a trial-specific charter based on the template created by the Damocles Group meeting at least annually. An emergency meeting may also be convened if a safety issue is identified. The DMC will report directly to both the TMG who will convey the findings of the DMC to the TSC and funders/sponsor as appropriate or when specifically requested by these parties.

#### Confidentiality

Confidential information collected during the trial will be stored in accordance with the General Data Protection Regulation 2018. As specified in the patient information sheet and with the patients’ consent, participants will be identified using only their date of birth and unique trial ID number. Authorised staff may have access to the records for quality assurance and audit purposes. The Trial’s Office maintains the confidentiality of all participants’ data and will not disclose information by which participants may be identified to any third party other than those directly involved in the treatment of the participant and organisations for which the participant has given explicit consent for data transfer (eg, NHS Digital, laboratory staff).

#### Trial status

Recruitment opened in July 2023 and is expected to last until 28 February 2027.

## Ethics and dissemination

The trial will be conducted in accordance with the recommendations guiding physicians in biomedical research involving human subjects, adopted by the 18th World Medical Association General Assembly, Helsinki, Finland and stated in the respective participating countries laws governing human research and Good Clinical Practice. The protocol (V.2.0, 7 July 2025) was approved on 6 August 2025 by West Midlands-South Birmingham Research Ethics Committee (ref: 22/WM/0230). Participants will be required to provide written informed consent. The IIH Intervention Trial’s Office will coordinate and communicate protocol modifications and modifications to all relevant parties.

A meeting will be held after the end of the study to allow discussion of the main results among the collaborators prior to publication. The results of this trial will be disseminated through national and international presentations and peer-reviewed publications. A lay summary of the results will be published online and provided to trial sites to share with participants.

Manuscripts will be prepared by the TMG and authorship will be determined by mutual agreement.

## Discussion

### Justification of trial design

IIH is a rare disease; hence, the numbers to be recruited are relatively small in the context of a phase IIb trial, but similar to other randomised controlled trials in IIH.^2 3 15^ A trial design with repeated measures analyses is, therefore, optimal for this rare disease setting as the models will use data from all time points, thus enabling a smaller sample size while maintaining power to detect a minimum clinically important difference.^41^

Patients will be randomised on a 1:1 ratio of intervention arms to provide an unbiased comparison and to avoid the potential confounding factors implicit in non-randomised comparisons. Randomisation will be minimised based on three key prognostic factors: (1) presence of or intention to insert a CSF lumbar drain prior to the intervention, (2) duration of IIH diagnosis and (3) degree of papilloedema. These factors were identified as prognostic for patient outcomes in a 9-year longitudinal cohort study of 490 patients with IIH.^49^ Baseline imbalances in these variables could influence trial outcomes and interpretation; therefore, minimisation, a dynamic randomisation method that ensures balanced allocation across groups based on selected baseline characteristics, is being used.

In addition to the virtual visit after 18 months of follow-up, two early visits (within the first 6 months) were permitted to be virtual in the updated version of the protocol (V.2.0, dated 7 July 2025). During the first 12 months of the trial being open, the ophthalmology burden on sites was preventing them from approaching patients for inclusion. Therefore, to ease this, sites were allocated two of a potential three visits (at months 2, 3 or 4). Sites are stratified by anticipated number of participants to be recruited to maintain an even distribution of in-clinic and virtual visits across these three time points. It is not expected that a site performs ophthalmology assessments during the virtual visits, and only a streamlined selection of the quality-of-life questionnaires are required.

The decision to group LP and VP shunts within a single ‘CSF shunt’ comparator arm was deliberate and based on both methodological and pragmatic considerations. First, current clinical practice for treating patients eligible for this trial varies across centres, with some institutions using LP shunts and others VP shunts. Mandating a single shunt type or requiring centres to randomise across three discrete surgical procedures would have required substantial deviation from established local practice. This may have limited site participation, impeded recruitment and reduced the generalisability of trial findings. Second, although there are differences between LP and VP shunts, both techniques represent accepted standard-of-care CSF diversion procedures and are used interchangeably in routine management of this condition. The primary objective of the trial is to compare DVSS with standard CSF diversion as practised in contemporary clinical settings, rather than to compare DVSS with a single prespecified shunt subtype. For this reason, preserving the heterogeneity inherent in real-world shunting practice reflects clinical reality and enhances external validity. As noted in the ‘Statistical analysis plan’ section, a subanalysis of each type of shunting may also be performed.

### Change in primary outcome

The choice of primary end point to determine successful outcomes in IIH has varied in the literature, including ICP, measures of papilloedema and visual function.^41 50^ Currently, there is no internationally agreed core outcome measurement set for trials in IIH.^51^ Papilloedema is a reliable sign of raised ICP in IIH.^40 52^ Change in papilloedema has been used by all previously published randomised controlled trials in IIH to date to determine clinical improvement.^43 53–55^

OCT imaging is an objective measurement routinely used to monitor patients with IIH and is recommended by professional bodies and the literature as both a diagnostic tool and to aid the longitudinal monitoring of papilloedema.^12 17 49 56^ Data acquisition with OCT imaging is rapid, highly reliable and reproducible, making it suited to patients presenting acutely with IIH. OCT measures of papilloedema in IIH are anatomical end points and data are emerging that they correlate closely with visual function.^40 57^

OCT measurements have also been found to reflect degrees of papilloedema and the ICP (as measured by LP). For example, the peripapillary RNFL thickness as measured by OCT has been shown to correlate with PMD in IIH.^57^ In the IIH treatment trial, the OCT parameters of RNFL, total retinal volume and optic nerve head volume were significantly correlated with both Frisén grades and ICP levels (as measured by LP).^58^

We acknowledge that optic nerve atrophy and reduction of papilloedema both cause a reduction in the global RNFL. Therefore, we will seek to differentiate this by monitoring and reporting the macular GCL. Further analyses to differentiate these are given in the SAP.

In the initial version of the protocol (V.1.0), PMD as measured using HVF over 12 months was defined as the primary outcome. However, there are a number of significant limitations to using PMD.^59 60^

Although HVF testing is routinely used in clinical practice to monitor visual function in IIH, measurement of PMD is not always reliable.^59 61^ The test is subjective and susceptible to variability related to headache burden, mood and patient understanding, often necessitating repeated assessments to achieve interpretable data. The IIH treatment trial highlighted this limitation, reporting that up to 21% of participants failed to complete at least one visual field satisfactorily.^62^ Poor test performance may be partially attributable to elevated ICP,^9^ a challenge particularly exacerbated in the IIH Interventional trial due to more severe disease at baseline compared with previous IIH trials.^43 55 63–65^ To mitigate the limitation of poor performance, prior studies in patients with IIH with milder disease have permitted up to four visual field attempts.^65^

While field repetition was allowed in our study, practical constraints (eg, urgency of surgical intervention or NHS resource limitations) may preclude repeat testing. Each test requires 30–45 min, which may be impractical in a clinical setting. As the primary outcome relies on data collected at all study visits, and the sample size calculation depends on the number of analysable fields, unusable tests contribute to missing data; this is particularly problematic if occurring at baseline.

Additionally, PMD shows a learning effect over time. In patients with established IIH, this is typically minimal due to prior exposure to HVF. However, newly diagnosed patients, as may be included in the IIH Intervention trial, may demonstrate test improvements attributable to learning rather than true improvement.

The PMD is also bounded above (≤0 dB) and effectively bounded below, as minimal improvement is observed in those with baseline PMD less than −10 dB. This challenges the assumption of PMD as a truly continuous outcome and may compromise statistical performance compared with genuinely continuous metrics.

Finally, cognitive impairments reported in IIH, such as poor concentration and slowed processing speed, can directly impact visual field reliability.^9^

Due to these limitations, the primary outcome was revised from PMD to global RNFL thickness, as assessed by OCT, measured over a 6-month period.

### Local regulatory governance

Within this IIH Intervention trial, the medical devices for CSF shunting are used according to their current indications-for-use (IFU). However, there is no licensed medical device available for stenting a dural venous sinus and the stents for DVSS will be used outside their current IFU. In centres where DVSS is currently routine practice, stents are already used off-label, following approval by individual Hospital Trusts. Following communication with the UK’s competent authority (Medicines and Healthcare products Regulatory Agency), this trial does not meet the criteria of a medical device study and is, therefore, not subject to national review by them. Therefore, participating institutions that currently do not perform DVSS will need to obtain the appropriate local governance approval for off-label use of individual medical devices used in DVSS intervention under new indication regulatory procedures applicable to each institution.

If this internal approval has already been obtained prior to the specific capacity and delivery assessment review performed by each NHS Trust, it should be incorporated into the assessment and subsequent R&D NHS institution approval process. It is expected that each trust and local clinician accept the indemnity and clinical responsibilities associated with the interventions detailed in this trial protocol. Additional information regarding these local governance issues will be included in the individual site agreements between the sponsor and NHS Trust.

As an additional layer of oversight, IIH Intervention has incorporated a DVSS accreditation committee. The role of the DVSS accreditation committee (experts in interventional neuroradiology who are independent of the sponsor) is to certify sites to be able to perform DVSS; review qualifications and experience for any new interventional neuroradiologist joining a clinical site after site opening before they are approved to perform DVSS within the trial and obtain and review confirmation from sites that local governance approval is in place for the use of stents outside their IFU. Proctoring of new interventional neuroradiologist is also in place to maintain procedural safety and quality.

## Supplementary material

10.1136/bmjopen-2025-111013 online supplemental file 1
