# Supplementary material for: Cerebrospinal fluid shunting or dural venous sinus stenting to preserve vision in idiopathic intracranial hypertension (IIH Intervention): protocol for an open-label, multicentre, randomised controlled phase IIb trial
Source: BMJ Open. 2026 Aug 3;16(8):e111013. doi: 10.1136/bmjopen-2025-111013 (PMC13436069; doi:10.1136/bmjopen-2025-111013)
Supplement: Supplementary file 1 [file bmjopen-16-8-s001.pdf]

Appendix 1: IIH Intervention trial schedule of events

	Baseline/ Randomisation	Intervention	Follow-up									End of trial	Rescue therapy /device failure
**Visit**	1	2	3	4	5	6	7	8	9	10	11	12	Unscheduled[14]
**Time (Week / Month)**			Wk 1	Wk 2	Mo 1	Mo 2	Mo 3	Mo 4	Mo 6	Mo 12	Mo 18	Mo 24	-
**Day**	-14 to 0	0	7	14	30	60	90	120	180	360	540	720	-
**Visit window (days)**	-	-	+/-3	+/-3	+/-7	+/-7	+/-7	+/-7	+/-7	+/-14	+/-30	+/-30	-
**Location**	Site	Site	Site	Site	Site	Site/ virtual[24]	Site/ virtual[24]	Site/ virtual[24]	Site	Site	Virtual	Site	Site
Inclusion/Exclusion criteria	x	x[15] (review)											
Informed consent	x												
Demography: (ethnicity, sex, DOB, first part of postcode)	x												
Medical, IIH and ophthalmic history	x												
Physical examination incl. Vital signs	x												x[18]
Height	x												
Weight and body mass index (BMI)	x		x	x	x	x	x	x	x	X		x	x
Urine Pregnancy Test (HCG)[1]	x												x[18]
Research blood sample collection	x									x			
Randomisation[2]	x												
LogMAR visual acuity testing[3, 23]	x		x	x	x	x	x	x	x	X		x	x[18]
Humphrey Visual Field (HVF)[4, 23]	x		x	x	x	x	x	x	x	X		x	x
Optical Coherence Tomography (OCT)[5,23]	x		x	x	x	x	x	x	x	X		x	x
Fundoscopic assessment of Papilloedema Frisén grade	x												
Imaging	(x)[6]	x[20]	x[21]										x[18, 22]
Intervention[7]		x											
Technical success evaluation[8]		x											
Evaluation of telemetric ICP (if applicable)		x	x		x		x		x	X		x	x

	Baseline/ Randomisation	Intervention	Follow-up									End of trial	Rescue therapy /device failure
**Visit**	**1**	**2**	**3**	**4**	**5**	**6**	**7**	**8**	**9**	**10**	**11**	**12**	**Unscheduled[14]**
**Time (Week / Month)**			**Wk 1**	**Wk 2**	**Mo 1**	**Mo 2**	**Mo 3**	**Mo 4**	**Mo 6**	**Mo 12**	**Mo 18**	**Mo 24**	-
**Day**	**-14 to 0**	**0**	**7**	**14**	**30**	**60**	**90**	**120**	**180**	**360**	**540**	**720**	-
**Visit window (days)**	-	-	**+/-3**	**+/-3**	**+/-7**	**+/-7**	**+/-7**	**+/-7**	**+/-7**	**+/-14**	**+/-30**	**+/-30**	-
**Location**	**Site**	**Site**	**Site**	**Site**	**Site**	**Site/ virtual[24]**	**Site/ virtual[24]**	**Site/ virtual[24]**	**Site**	**Site**	**Virtual**	**Site**	**Site**
Headache history	x												
Review headache over past month[9]	x												x
Headache diary provision[10]		x			x	x	x	x	x	X	x		
Review of headache diary completion			x	x	x	x	x	x	x	X	x[17]	x[17]	x
Headache disability (HIT-6)	x				x[16]	x[16]	x[16]	x[16]	x[16]	x[16]	x[16]	x[16]	x[19]
SF-36v2	x				x[16]	x[16]	x[16]	x[16]	x[16]	x[16]	x[16]	x[16]	x[19]
Tinnitus questionnaire (THI)	x	x	x	x[16]	x[16]	x[16]	x[16]	x[16]	x[16]	x[16]	x[16]	x[16]	x[19]
NEI-VFQ-25 with 10-Item Neuro-Ophthalmic Supplement	x	x	x	x[16]	x[16]	x[16]	x[16]	x[16]	x[16]	x[16]	x[16]	x[16]	x[19]
QoR-15[11]		x											
Satisfaction with Intervention[11]		x											
WPAI-GH	x					x[16]			x[16]	x[16]	x[16]	x[16]	x[19]
EQ5D-5L	x		x		x[16]			x[16]	x[16]	x[16]	x[16]	x[16]	x[19]
Healthcare resource use questionnaire	x		x		x[16]			x[16]	x[16]	x[16]	x[16]	x[16]	x[19]
Adverse Event data review[12]		x	x	x	x	x	x	x	x	X	x	x	x
Concomitant Medications review	x	x	x	x	x	x	x	x	x	X	x	x	x
NHS Digital data acquisition[13]												x	

DOB = Date of Birth, HCG = Human Chorionic Gonadotrophin, LogMAR = log of the minimum angle of resolution, CTV = Computed Tomography Venography, MRV = Magnetic Resonance Venography, HIT-6 = Headache Impact Test, SF-36v2 = 36-item Short Form Survey (version 2), THI = Tinnitus Handicap Inventory, NEI-VFQ-25 = National Eye Institute Visual Functioning Questionnaire 25, QoR-15 = Quality of Recovery-15, WPAI-GH = Work Productivity and Activity Impairment Questionnaire – General Health, EQ5D-5L = EuroQol - 5 Dimensions- 5 Levels.

Note: Where visits 1 and 2 are performed less than 24 hours apart, the assessments as part of visit 1 must be performed prior to the assessments at visit 2.

Key:

1. Women of childbearing potential only.
2. Randomisation to be performed within 14 days from date of consent and only if intervention is also expected to be within 14 days of consent.
3. Recording visual acuity using the LogMAR chart.
4. Humphrey Visual Field (HVF) Analyser using 24-2 Swedish Interactive Threshold Algorithm (SITA) standard test pattern using a size III white stimulus. Sites should check the performance of the HVF (aiming for no more than 15% false positives).
5. Spectral Domain optical coherence tomography (OCT) imaging for retinal nerve fibre layer thickness, optic nerve head size, and macular ganglion cell layer / complex thickness.
6. Previous computed tomography venography (CTV) or magnetic resonance venography (MRV) can be used provided it was performed within 12 weeks prior to intervention.
7. Intervention (cerebrospinal fluid [CSF] shunting or dural venous sinus stenting [DVSS]) to be performed after randomisation and within 14 days of consent.
8. Technical success evaluation performed locally for CSF shunt and for DVSS based on peri-/post-operative imaging.
9. Review of headaches over the past 28 days. If patient does not have records up to 28 days, as a minimum a review of headache over at least the past 7 days should be recorded. If a lumbar puncture (LP) has been conducted during the period of the headache assessment the details of the headache in the 7 days prior to the LP should be used.
10. Participants will be provided with a headache diary at Visit 2 and will be given instructions for its use. Diary should then be completed daily up to Visit 10, and then daily for 28 days before Visit 11 and Visit 12. Between Visit 10 and Visit 12, participant should restart diary completion if feeling unwell.
11. Questionnaire to be completed on day of discharge.
12. Adverse events (AE) and complications data will be collected according to Clavien-Dindo classification of surgical complications [47] and CTCAE v5.0 [48] during the AE reporting period (consent to Day 360). If there is any Serious AE (SAE) related to the medical device after 12 months, this will be reported up to 24 months post trial intervention.
13. Remote collection from NHS Digital of patient health data routinely collected, such as (but not limited to) hospital admissions and diagnostic imaging, from intervention to 24 months (and up to 10 years post intervention).
14. For unscheduled visits related to rescue therapy/device failure query, if it is an emergency, the site clinician can proceed with the therapy. If it is not an emergency, the site clinician should repeat the ophthalmology assessment and ask the Independent Adjudication Committee to review the ophthalmology data before making a decision.
15. This is a safety check prior to intervention.
16. Questionnaire to be completed by a research nurse during or prior to the clinic visit. For virtual visit (V11 + two of V6, V7 & V8 as per pre-specified site allocation), All questionnaires to be administered over the phone during the visit except SF36v2, Tinnitus Questionnaire (THI) and NEI-VFQ-25 with 10-Item Neuro-Ophthalmic Supplement.

17. Nurse telephone call 1-month beforehand to restart daily headache diary completion.
18. As clinically indicated. Pregnancy test to be repeated prior to any revision of an intervention and prior to any repeated imaging involving radiation as per local practice.
19. If not completed in the previous 8 weeks.
20. Imaging to be performed according to local practice at site for the trial intervention, please refer to the trial DVSS and CSF shunting SOPs.
21. One CTV or MRV as follow-up imaging for DVSS arm will be performed according to local practice at site (please refer to the trial DVSS SOP). The timing will vary according to local practice at site. It will be within 12 months of intervention.
22. Imaging will be performed according to the emergency local procedure in place at site. In case of suspected failure of the intervention and subsequent revision, imaging will be performed according to the type of revision intervention decided by the trial investigator or responsible consultant and according to local practice.
23. Depending on individual site allocation, where these visits are performed virtually, the following ophthalmology assessment may not need to be performed at two of visits 6,7 & 8 depending on individual site allocation. All other assessment are required to be performed except the questionnaires specified. See protocol visit schedule section for additional information.
24. Two of visits 6, 7 and 8 may be virtual visits depending on individual site allocation prescribed in Trial Specific guidance.

Appendix 2: WHO trial registration data set for the IIH Intervention trial

**Data category**	**Information**
Primary registry and trial identifying number	ISRCTN: 57142415
Date of registration in primary registry	02-Feb-2022
Secondary identifying numbers	n/a
Source(s) of monetary or material support	National Institute for Health Research Health Technology Assessment, NIHR HTA (reference NIHR131211)
Primary sponsor	University of Birmingham
Secondary sponsor(s)	n/a
Contact for public queries	IIHIntervention@trials.bham.ac.uk
Contact for scientific queries	a.b.sinclair@bham.ac.uk
Public title	IIH Intervention: A clinical trial comparing two treatments (shunts and stents) to preserve vision for people with idiopathic intracranial hypertension
Scientific title	Cerebrospinal fluid shunting or dural venous sinus stenting to preserve vision in idiopathic intracranial hypertension (IIH Intervention)
Countries of recruitment	UK
Health condition(s) or problem(s) studied	Idiopathic intracranial hypertension
Intervention(s)	Arm 1: Cerebrospinal fluid shunting<br>Arm 2: Dural venous sinus stenting

Data category	Information
Key inclusion and exclusion criteria:	Ages eligible for study: Aged 18 to <64 years<br>Sexes eligible for study: Both<br>Accepts healthy volunteers: No
	Inclusion criteria: Confirmed idiopathic intracranial hypertension (IIH); papilloedema (Frisén grade ≥ 3) in at least one eye; able to provide written informed consent
	Exclusion criteria: Venous sinus thrombosis; bariatric surgery within previous 3 months; previous surgery for IIH; other causes of raised intracranial pressure; pregnancy
Study type	Interventional
	Allocation: Two-arm, randomised, open-label trial
	Primary purpose: Efficacy
	Phase IIb
Date of first enrolment	17-Nov-2023
Target sample size	80 (randomised 1:1)
Recruitment status	Open
Primary outcome(s)	Global thickness of retinal nerve fibre layer (RNFL), as measured using optical coherence tomography (OCT), over 6 months
Secondary outcome(s)	OCT measures over 12 and 24 months<br>• Global thickness of the retinal nerve fibre layer (RNFL)<br>OCT measures at 6, 12 and 24 months<br>• Total retinal thickness<br>• Disc global volume<br>• Disc central thickness<br>• Disc maximum height<br>• Macular ganglion cell layer volume

<table>
<tr><th>Data category</th><th>Information</th></tr>
<tr><td></td><td>
Visual acuity, measured by logarithm of the minimum angle or resolution (LogMAR) units over 6, 12 and 24 months.
HVF Perimetric mean deviation (PMD) over 6, 12 and 24 months.
Proportion of participants re-presenting to hospital within 30 days.
Proportion of participants being re-admitted to hospital within 30 days.
Outcomes to be recorded over 6, 12 and 24 months:

Proportion of major complications, defined as the incidence of complications graded between III and V or major anaesthesia problems (including complications from revision procedures) according to the Clavien-Dindo classification of surgical complications
Proportion with minor complications, defined as Clavien-Dindo grade I-II during hospital stay
Proportion of participants requiring revision in whom:

the revision intervention is the same as the primary intervention
the revision intervention crosses over to the alternative intervention

Time to first revision
Number of revisions per participants
Proportion of participants with adverse events grade 3 or above as measured by the CTCAE version 5

Headache outcomes to be recorded over 6, 12 and 24 months

Monthly headache days
Monthly moderate to severe headache days
Headache severity (numeric rating scale (NRS) 0-10)
Moderate to severe headache severity (NRS ≥ 4)
Monthly use of acute headache analgesics

Patient Reported Outcome Measures (PROM)

Quality of Recovery 15 (QoR-15) measured at time of discharge
Satisfaction with intervention (5-point Likert scale) measured at time of discharge.

Other outcomes to be recorded over 6, 12 and 24 months:

Visual Function Questionnaire (NEI-VFQ-25 with 10-Item Neuro-Ophthalmic Supplement)
Headache Impact Test (HIT-6)
36-item Short form health survey (SF-36v2)

</td></tr>
</table>

Data category	Information
	• EuroQol 5 dimension 5 level survey (EQ5D-5L)<br>• Time to return to work (if working)<br>• Work Productivity and Activity Impairment Questionnaire – General Health (WPAI–GH)<br>• Proportion of participants requiring ICP lowering medication above a clinically relevant threshold<br><br>Healthcare resource use questionnaire

Appendix 3: Exemplar IIH Intervention trial patient information sheet

Participant Information Sheet

IIH Intervention: A clinical trial comparing 2 treatments (shunts and stents) to preserve vision for people with Idiopathic Intracranial Hypertension (IIH)

WE WOULD LIKE TO INVITE YOU TO TAKE PART IN A CLINICAL TRIAL

This is a non-commercial clinical trial funded by the National Institute for Health Research (grant number: NIHR131211) and sponsored by the University of Birmingham, Edgbaston, Birmingham B15 2TT (the Sponsor) – IIH Intervention trial.

This information sheet is designed to help you understand what the trial is about. Taking part in this trial is voluntary, and before you decide it is important for you to understand why the research is being done and what it will involve. Please read and discuss it with anyone you wish. You may take this sheet away with you. Do feel free to ask if there is anything that is not clear or if you would like more information. Take time to decide whether or not you wish to take part.

This information sheet has been split into two parts:

- **Part one** tells you why we are doing this trial and gives information about the 2 treatments (shunts and stents)
- **Part two** tells you what will happen if you take part and gives you more detailed information about the trial.

Please ask us if there is anything that is not clear.

IMPORTANT THINGS THAT YOU NEED TO KNOW

This trial is for adults and not children. All adults who take part in this trial have a diagnosis of Idiopathic Intracranial Hypertension (IIH) and are at risk of permanent sight loss.

In this trial, the researchers want to compare two treatments (shunting and stenting) to find the best treatment to save a person's eyesight. We also want to find out which treatment is the best in the long term.

This Participant Information Sheet is provided to help you understand what treatment will be given and will provide answers to the questions on the right of this page.

Thank you for reading this Participant Information sheet and considering the IIH Intervention trial.

CONTENTS

Part One:

1. What is the purpose of this clinical trial?
2. Why have I been invited to take part?
3. What do I need to know about the two types of treatment used in this trial?
4. Do I have to take part?

Part Two:

5. What does taking part involve?
6. What are the potential benefits?
7. What are the possible risks?
8. Pregnancy and potential harm to an unborn baby
9. More information about taking part
10. Confidentiality
11. Further information and contact details

PART ONE: What is this trial about?

1. WHAT IS THE PURPOSE OF THIS CLINICAL TRIAL?

Idiopathic intracranial hypertension (IIH) is a rare condition of unknown cause that results in raised intracranial (brain) pressure. People with IIH typically present with visual disturbance (e.g. blurred vision or sudden darkening of vision) and headache.

Some people with IIH are at risk of permanent sight loss and require emergency intervention to lower brain pressure in order to prevent permanent loss of sight.

We are doing this trial because we want to compare two treatments (shunting and stenting), to find the best treatment to save eyesight in people with IIH at risk of permanent sight loss. The research is designed to find out which treatment is the best in the long term.

The two treatments that will be compared are:

1. **Cerebrospinal fluid (CSF) Shunting** (shunting) – A shunt is a narrow flexible silicon tube with one end inserted in the CSF space in the brain (ventricle) or lower spine and the other end into the abdominal cavity, to allow drainage of CSF and to lower the brain pressure.
There are two types of shunts: a ventriculoperitoneal (VP) shunt where the tube is inserted into a part of the brain called the ventricle and a lumboperitoneal (LP) shunt where the tube is inserted in the lower part of your back (spine). Both types of shunts are available, but the choice of shunt will be decided following discussion with the surgeon and according to local practice. One of the limitations of a VP shunt is that individuals are not allowed to drive for 6 months after the operation.

2. **Dural Venous Sinus Stenting** (stenting) – A tube made of metal mesh (stent) is passed through the vessels into a narrow part of one of the large drainage veins in the brain, in order to widen it. The stent is inserted through a small skin incision via the veins using a small tube (catheter) and wire, under X-ray guidance.

We also refer to shunting and stenting treatments as interventions.

For this trial, one half of the participants will randomly have a shunt, and one half will have a stent. This is what we call '**randomisation'**. The chance of having either intervention is the same. It is important that you accept that the decision whether you will have a shunt or stent is decided in a random way. It is important that you understand that the doctor has no influence on whether you are randomised to receive a shunt or a stent. Randomisation is important because it makes sure that every person has equal chance of receiving any of the two treatments and makes the results fair.

Participants are then followed up with regular assessments. Participants will be seen by the hospital/ doctor/ care team regularly to have some tests and see how they are feeling.

Participants will also be asked to give us permission to access some of their medical data from NHS digital (these are NHS electronic health records) for up to 10 years after their intervention. The researchers will use this data for long term follow-up rather than additional trial appointments.

2. WHY HAVE I BEEN INVITED TO TAKE PART?

You have been approached because you have been diagnosed with IIH and need emergency treatment to lower brain pressure and save your eyesight.

3. WHAT DO I NEED TO KNOW ABOUT THE TWO TYPES OF TREATMENT USED IN THIS TRIAL?

Ventriculoperitoneal (VP) and Lumboperitoneal (LP) Shunts:

VP and LP shunts drain CSF from the brain or lower spine to the abdominal cavity and this immediately reduces brain pressure.

For a VP shunt, one or two small cuts are made in the scalp and a small hole is drilled into the skull beneath the cut. A thin tube (catheter) is then placed into a fluid-filled space of the brain called the ventricle to drain CSF. The other end of the tube is then placed under the skin, from behind the ear, down the neck and chest to the abdomen. Another one or two small cuts are made in the abdomen and the catheter is placed inside the abdomen to drain the CSF. The cuts will be closed with stitches. The tube has a valve (a control device like a tap) that regulates the flow of fluid and in some cases may have a small reservoir or a pressure monitor to help with the function and the assessment of the shunt in cases of suspected malfunction. The valve may be adjustable, which means that the drainage of fluid can increase or decrease by changing the setting of the valve.

For an LP shunt, one end of the catheter is placed in the CSF (fluid) space at the bottom of the spine through a skin cut at the lower back and the other end is placed in the abdomen, through one or two more skin cuts. The shunt drains the excess fluid from the spine, which communicates with the fluid in the brain, into the abdomen. The shunt has a valve that regulates the flow of fluid and this valve may also be adjustable.

Both types of shunts are inserted in the operating theatre under general anaesthetic. The type of shunt that you will have will be decided following discussion with the surgeon (see above). A shunt remains in the body for ever. It is only removed if it causes problems like infection.

As with any surgery there are risks of complications – please see **section 7** for full details.

There are a few professions that exclude people with shunts, such as the UK armed forces, due to the demands and unpredictability of the job.

If you have a VP shunt placed and drive in the United Kingdom (UK) then you will have to notify the DVLA of this and will not be allowed to drive for 6 months. If you suffer a seizure, you will not be able to drive for longer.

Stenting:

Stenting is an emerging procedure for people with IIH and is currently routinely offered at a limited number of centres in the UK.

Depending on local practice, before and after the procedure, blood thinning tablets are given to reduce the risk of developing a blood clot in the stent. In most centres the blood thinning tablets are continued for 6 months after the procedure.

The stenting is performed in the Radiology Department under local or general anaesthesia. A thin tube (catheter) and wire are inserted via a large vein in either the groin or neck. The wire is then passed through the blood vessels to the main veins of the brain (dural venous sinus) inside the head. From here, the doctors are able to use X-rays and a special dye to show the narrowed part of the vein. A stent (wire mesh tube) is then introduced via the catheter and delivered across the narrowing of the vein in order to widen it and keep it open. The stent remains in the body for ever.

Please see section 7 for the list of potential complications.

If you take part in the trial, you will be carefully monitored for any complication, and will be offered further treatment if necessary, which may include another stenting or shunting.

4. DO I HAVE TO TAKE PART?

No. Participation is voluntary. If you decide to take part, you will be asked to sign a consent form and will be given a copy to keep. You may withdraw from the trial at any time and you do not have to give any reason for your decision.

If you decide not to take part, your doctor will continue to treat you with the best means available and the standard of your care will not be affected.

If you choose to take part in the trial but later choose to withdraw, we would still like to collect information about your treatment as this will be invaluable to our research. If you have any objection to this please let your doctor know. Data already collected prior to withdrawal will be kept and analysed.

Thank you for taking the time to read Part One.

If you are interested and are considering participation, please read the additional information in Part Two before making any decision.

PART TWO: What do I need to know before I take part in this research?

5. WHAT DOES TAKING PART INVOLVE?

If you agree to take part in this study you will be provided with a copy of this information leaflet and will be given the opportunity to discuss the study with a member of your local clinical team. You will be given time to decide whether you wish to participate in the trial. During this time, you may want to discuss this with your friends or family.

If you decide to take part you will be asked to sign a consent form and will be given a copy to keep. Following this, you will be asked to attend 9 hospital appointments and 3 telephone appointments. Your trial participation will take 2 years from start to finish. There is a **chart on p.11** with the details of all the visits and assessments.

With your permission, the sponsor will also obtain routinely collected health data from NHS Digital (the National custodian of NHS health and social care data) about your health and any other hospital appointments you may have for up to 10 years after the trial intervention. There are no trial appointments between 2 years and 10 years.

Visit 1 - Screening visit.

This visit may last between 1 and up to 14 days. During this visit your doctor will first check your eligibility by asking relevant questions and performing tests, including a pregnancy test (if female). During your evaluation of sight loss due to IIH, you will require brain imaging and this will be done according to your hospital standard of care, irrespective of whether you participate in the trial or not. Once your doctor has confirmed that you are potentially eligible for the trial, you will be invited to participate. Your participation is voluntary and if you agree, you will be asked to sign a consent form to indicate your agreement. If you agree to participate you will then be allocated a screening number. With your permission, your local clinical team will collect 8ml of blood (approximately 2 teaspoons) for future research, on top of the routine blood tests you will have before the intervention. You will also be asked to complete questionnaires. After all your trial investigations have been received and your eligibility to the trial has been confirmed, you will be randomly allocated to either the Shunting group or the Stenting group and will be allocated a trial number. This random allocation is done by a computer and not a member of the Trials Office or a doctor and is called **randomisation**.

Visit 2 – **Intervention and post-intervention admission**.

This visit is from the date of the procedure (shunting or stenting) to the date of discharge from hospital. You will have the procedure within 14 days of consent. You may have scans or X-rays before and after the procedure, depending on the intervention that you will have and local hospital standard of care. You will be monitored for any complications and you will stay in hospital for about 1-2 days after the intervention. Your local clinical team will also ask you to complete questionnaires. You will also receive a headache diary and be explained how to use it.

The diary will just take a few minutes to complete each day. You will be asked to complete it **every day for the first 12 months**, and to complete it for about 30 days before visit 11 and visit 12. After the first year, you should also complete it if you are feeling unwell. The headache diary is the only commitment between assessments.

Visit 3-10 – follow-up visits.

These visits are designed to check your general health, do eyesight tests and complete questionnaires to determine what effect the intervention has had. Six of these visits will be in-person, two may be virtual/telephone appointments.

These visits will be done 1 week (visit 3), 2 weeks (visit 4), 1 month (visit 5), 2 months (visit 6), 3 months (visit 7), 4 months (visit 8), 6 months (visit 9) and 12 months (visit 10) after the day you had your intervention. For each in-person visit, assessments are expected to take approximately 2 hours and questionnaires will take between 30min and 1 hour to complete. For virtual appointments, your local clinical team will telephone you to ask you some questions about your health. Headache diary review, and questionnaires will take up to 1 hour to complete.

During each in-person visit, your local clinical team will check that you are well and check your vision. We will also ask you to bring your completed headache diary. You will be given a new diary and will be asked to complete it every day until your next visit and bring it at your appointment. We will ask you to complete the diary every day during the first 12 months.

For the visit 10 (12 months), with your permission, we will collect 8ml of blood (approximately 2 teaspoons) for future research.

At each visit, your local clinical team will ask you to complete a set of questionnaires. You will have the option to complete the questionnaires at home before your appointment. If you request this, your local clinical team will provide the questionnaires either at your previous visit, or post them to your home address (or with your permission email them to you if preferred). We will ask you to bring the completed questionnaires to your hospital appointment.

If during a visit, we suspect that your shunt or the stent is not functioning properly, you may have further investigations based on your local hospital practice.

Visit 11 – virtual/telephone follow-up appointment.

Your local clinical team will telephone you in between your yearly hospital visits to ask you some questions about your health. This virtual appointment will be done 18 months after your initial intervention.

Your local clinical team will contact you about 30 days before your appointment and will ask you to complete your headache diary **every day until your appointment**. They will review your completed headache diary over the phone with you during the appointment.

Your local clinical team will ask you to complete a set of questionnaires at home before your appointment. They will post the questionnaires to your home address (or with your permission email them to you if preferred) and will ask you to read out your answers during the appointment. The remote follow up appointment will take up to 1 hour to complete if you answered the questionnaires before the visit, or up to 2 hours if you are completing them while on the phone.

Visit 12 – final follow-up visit.

This final visit will be done 24 months after your initial trial intervention. Your local clinical team will check your general health, do eyesight tests and ask you to complete questionnaires to determine what effect the intervention has had.

Your local clinical team will contact you about 30 days before your appointment and will ask you to complete your headache diary every day until your appointment. You will be asked to bring your completed headache diary to the appointment.

Your local clinical team will ask you to complete a set of questionnaires. You will have the option to complete the questionnaires at home before your appointment. In that case, you will need to bring the completed questionnaires to your hospital appointment. The assessments will take approximately 2 hours to complete and questionnaires will take about 1 hour to complete.

If your local clinical team feel at any point that you need to be seen by a doctor more frequently, they can add in additional appointments between visit 2 and visit 12.

Your trial participation will stop at Visit 12, which will be 2 years after you entered the trial.

Covid-19

To make any risks due to COVID-19 as small as possible, some of these assessments may be delivered remotely, in accordance with applicable government guidelines. Paper diaries and questionnaires may be shipped to your address (or emailed, if preferred) and reviewed remotely. This will, however, be dependent on the doctors who are treating you and your hospital procedures.

Imaging procedures

In order to check that the intervention has been successful, scans and X-rays may be performed, depending on the type of intervention.

After a VP shunt you will have a CT scan of the brain and X-rays of the shunt. After an LP shunt you will only have X-rays of the shunt. These are performed to make sure that the shunt is in the right place and is part of standard care, not related to the trial.

During insertion of the stent, X-ray pictures of the brain are taken during the procedure to guide the stent to the narrowing of the vein. After the placement of the stent, X-ray pictures will be taken again, to make sure that the stent is in the right place. This type of X-ray is called digital subtraction angiography. Another brain scan will be done within one year after the intervention. The timing and type of scan (CT or MRI brain) will depend on local practice.

For more information about radiation risks related to imaging procedures, please see **section 7**.

Review of visual tests, brain images and brain pressure recordings.

As part of the research process it is important that the same people are looking at your test results; this will help strengthen the results. This will mean that some of your tests, such as visual tests results and images, brain images (MRI and/or CT scan) and in some cases recordings from a brain pressure monitor of the VP shunt (if you have a VP shunt with this device) will be transferred to the University of Birmingham and/or to the lead site, University Hospitals Birmingham NHS Foundation Trust, for review by nominated centres of excellence. These images will be pseudo-anonymised (data linked only using your unique trial number, your name and personal details are not seen) before being submitted for review. These centres of excellence may be located in the United Kingdom (UK), European Union (EU) or outside the EU eg United States of America (USA), and we require your consent to allow these centres to access material for a "Central Review" for research purposes.

Optional Research blood samples

If your site has the equipment required, in addition to routine clinical samples, with your permission, your local clinical team will take and store 8ml of your blood (approximately 2 teaspoons) to perform future research on IIH.

Your local clinical team will take your blood in the same way that a regular blood test is taken. All blood tests may cause a momentary sharp pain on insertion of the needle and carry a risk of infection, which is extremely rare.

With your permission, these samples will be available for other medical research projects that have been approved by a Research Ethics Committee. Anyone using these samples for research will not have access to your personal details. It is difficult to predict exactly what scientific developments there may be so we cannot give precise details of what research might be done.

During and after the trial, you are the owner of the samples. This gives you the right to have any remaining sample material destroyed (by the Sponsor) at any time. If you choose to have any remaining samples destroyed please contact your trial doctor (see contact details on the front and last pages of this information sheet).

Whether or not you agree to the collection of research blood samples will not affect your participation in the clinical trial or your medical care.

Chart of trial visits and assessments:

Trial Visits	Visit 1 screening	Visit 2 intervention	Visit 3 Week 1	Visit 4 Week 2	Visit 5 Month 1	Visit 6 Month 2	Visit 7 Month 3	Visit 8 Month 4	Visit 9 Month 6	Visit 10 Month 12	Visit 11 Month 18	Visit 12 Month 24	Additional Visit
**Location**	**Clinic**	**Hospital ward**	**Clinic**	**Clinic**	**Clinic**	**Clinic or virtual**	**Clinic or virtual**	**Clinic or virtual**	**Clinic**	**Clinic**	**Virtual**	**Clinic**	**Clinic**
Eligibility assessments	X	(X)											
Consent	X												
Clinical assessment	X												(X)
Weight and body mass index (BMI)	X		X	X	X	X*	X*	X*	X	X		X	X
Pregnancy Test	X												(X)
Research bloods (optional)	X									X			
Randomisation	X												
Fundoscopy for papilloedema (swelling at the back of the eye)	X												
Visual tests (visual acuity, field, scan)	X		X	X	X	X*	X*	X*	X	X		X	X
Trial intervention (shunting or stenting)		X											
Imaging		X	ROUTINE POST-INTERVENTION IMAGING										(X)
Headache diary review		X	X	X	X	X	X	X	X	X	X	X	X
Questionnaires	X	X	X	X	X	X	X	X	X	X	X	X	(X)
Recording of brain pressure (For VP shunt only, if pressure device is used)		X	X		X		X		X	X		X	X
Adverse Event review	X	X	X	X	X	X	X	X	X	X	X	X	X
Medications review	X	X	X	X	X	X	X	X	X	X	X	X	X

*this assessment will not be performed if your visit is virtual.

The “Additional visit” would be needed if your local clinical team had any concerns and wanted to re-check you. In that case, they will ask you to come for a clinic visit and will perform some additional tests. A pregnancy test and additional imaging may be required if your doctor thinks you may need another intervention treatment. You may also be asked to complete questionnaires if you did not already complete them within the previous 8 weeks.

Eligibility Assessments: Your doctor will check that you can have either type of treatment (shunting or stenting).

Clinical Assessment: Your doctor/local clinical team will take your medical history (including headache history), and ask you questions about your health, any medication you have taken over the last 4 months, and any symptoms you have been experiencing. It will also include a physical examination and they will record your height. Your clinical assessment should take around 30 minutes to complete.

Pregnancy test (female participants only): This urine pregnancy test will be performed on all women of child-bearing potential. A nurse will test your urine sample with a dipstick and will tell you the result immediately.

Fundoscopy for Papilloedema: Fundoscopy is a test that allows your doctor to look at the back of your eye. Your doctor will use it to check your papilloedema (swelling at the back of the eye). Your doctor will put drops in your eyes and wait for 20 minutes for them to take effect. Then they will do the exam. The drops will keep your eye dilated for several hours, so your vision may be blurry during that time.

Visual tests: Your doctor/local medical team will test your eyesight at every clinic visit before and after the intervention. They will use a number of different techniques which test how large your field of vision is and how well you read the letters on a chart (visual acuity). They will also measure your papilloedema again using a scanning laser (OCT), routinely used in clinical practice. For these tests, your doctor may need to put drops in your eye to do the exam. The drops will keep your eye dilated for several hours so your vision may be blurry during that time. The visual tests will take around 1 hour to complete.

Headache diary: Your local clinical team will give you a headache diary to take home and complete with details of any headache you may have. It is important for us to monitor your headaches to determine which treatment is better. The first diary will be given after the intervention and your local clinical team will explain how to use it (visit 2). The diary will just take a few minutes to complete each day. You will be asked to complete a diary every day for the first 12 months, and to complete it for 30 days before visit 11 and visit 12, or if you are feeling unwell. You will need to bring your completed diary to each clinic visit so that your local clinical team can review it, and you may be given a new diary if needed.

Questionnaires: Your local clinical team will ask you to complete a set of questionnaires about your quality of life and your health-related expenses. These will include quality of life questionnaires, a tinnitus questionnaire, a headache questionnaire, questionnaires about the intervention, and questionnaires about your sight. At each visit, you will be asked to complete a set of paper questionnaires and you can ask for assistance if you need it. In total the questionnaire will take around 30min to 1h to complete (depending on the visit). For visits 3-10 and visit 12, you can complete the questionnaires during your assessments or ask to complete the questionnaires at home before your visit. If you have completed the questionnaires at home, you will need to bring them to your appointment.

Adverse events and medication review: Your doctor will perform an assessment of your current wellbeing, health, a record of other medication you are taking, and any symptoms you have been experiencing since your last visit. This will take around 10 minutes to complete.

6. WHAT ARE THE POTENTIAL BENEFITS?

There are no direct benefits to you from taking part in the trial but the information gained from this trial may help improve treatment for adults with IIH in the future. You may be seen more often and/or feel more supported as a consequence of your involvement in the trial.

This trial is intended to see which type of intervention is better to treat IIH when there is a sudden loss of sight and which is the most cost-effective.

7. WHAT ARE THE POSSIBLE RISKS?

As with any intervention there are risks and complications, but there are no additional disadvantages or risks involved in taking part in this trial. Both CSF shunting and stenting are treatments for IIH (shunting is widely used internationally, and in some hospitals, stenting is used as part of standard of care). You require an intervention to prevent sight loss. None of these treatments are experimental but at present there is not enough information to determine which treatment is most suitable and provides the higher level of health benefits to the individual.

For **CSF shunting**, very common complications (those that affect more than 1 in 10 people) include:

- Headache
- Pain in the neck, chest and abdomen, lower back and joints over the first few hours or days, which typically settles
- Temporary hearing symptoms
- Mechanical complications of the shunt (becoming blocked, disconnected or not working properly). This may require further surgery (about 1 in 4 people will have a revision within 1 month, about 1 in 2 will have a revision within 1 year).

Other common complications (those that affect between 1 in 10 to 1 in 100 people) are:

- Infection, like meningitis, or infection in other locations within the body
- Sciatica (leg pain from irritation of the nerves by the LP shunt)
- Dizziness (vertigo)
- Ringing in your ear (tinnitus)
- Vision symptoms
- Blood clot blocking a vein
- Minor pain and bruising from intravenous cannula site
- Bleeding (associated with medical device location)
- Seizures (only with VP shunts)

For **stenting**, a very common complication (affecting more than 1 in 10 people) is:

- Worsening of headache, over the first few hours or days which typically settles.

Other common complications include:

- Bruising in the groin or neck area (depending where the tube was inserted)
- Increased bleeding due to blood thinning medication
- New narrowing within the stent or elsewhere in the brain veins (sinuses).

There are also the risks associated with general anaesthesia and hospital admission, like severe allergic reactions, heart attack, urinary tract and chest infections (including COVID-19) and formation of blood clots in the legs and the lungs (pulmonary embolism).

Both shunting and stenting have rare complications that may lead to significant long-term-disability or even death. These include severe infections (e.g. brain abscess, peritonitis, blood infection), stroke and brain haemorrhage and inadvertent injury during the intervention (e.g. of a big vessel causing significant bleeding requiring transfusion).

Radiation risks

CT scans, imaging done during stenting (digital subtraction angiography) and X-rays all use radiations.

Imaging performed for CSF shunting (which can include CT scans or MRI scans, and X-rays of the shunt) are standard of care in all centres.

Whilst imaging done during stenting is standard of care in some centres, in others it is above standard of care and therefore may involve an additional dose of radiation.

If you take part in this study you may have CT Head procedures and X-rays taken during the insertion to guide the stent and after the insertion, to ensure that the stent is in the correct place. Some of these may be extra to those that you would have if you did not take part. These procedures use ionising radiation to form images of your body and provide treatment. Ionising radiation may cause cancer many years or decades after the exposure.

The chances of this happening to you as a consequence of taking part in this study is about 0.14%.

If you have a concern about any part of this trial, please speak to your doctor or one of the trial doctors. Your local clinical team will do their best to answer these questions (see contact details on last page). If you still have concerns, you can raise these using the NHS complaints procedure. You can ask how to do this at your hospital.

If something goes wrong while you are taking part in this Trial, your local clinical team will do everything they can to look after you and will make sure you are closely supervised while taking part in the Trial.

You are free to withdraw at any time without having to provide a reason.

If you decide to stop taking part in the trial, you will only need to inform your doctor and he or she will withdraw you from the trial. This will not affect your standard of care.

8. PREGNANCY AND POTENTIAL HARM TO AN UNBORN BABY

To participate to this trial, if you are female of childbearing potential, you should not be pregnant before the intervention. Pregnancy may exacerbate IIH. Typically whilst IIH is at risk of rapidly declining eyesight, pregnancy is not advised. This is irrespective of the intervention. The risks of pregnancy settles once the IIH has stabilised and you have recovered from the intervention.

If you become pregnant while you are in follow up and still participating to this trial, you should tell your doctor without delay.

If you require a repeat intervention or need to have imaging, you will be asked to do a pregnancy test, as some imaging procedures may be harmful to the unborn baby.

Your fertility will not be affected if you participate to this trial. Assisted fertility treatment and procedures will need to be evaluated on an individual basis according to your health status and the medication you are receiving. There is no impact on male fertility from participating in this trial.

9. MORE INFORMATION ABOUT TAKING PART

Does taking part in this trial affect the medication I am currently taking or will take in the future?

Whilst taking part in the trial you will be asked to let your doctors know if you are taking any other medicines.

Medications to treat headache are allowed, including headache preventative medications but if your doctor starts these you should inform your local clinical team at the next trial visit. Painkillers are permitted and if used for headache, please document this in your headache diary each time they are used.

Usually medications to reduce brain pressure (including Acetazolamide, Topiramate, diuretics, glucocorticoids (oral Dexamethasone and oral Prednisolone)) would be discontinued following the intervention. These can be given only if prescribed by your doctor as rescue medication and this needs to be checked with your local clinical team before they are started unless it is an emergency. If you have any questions about medications check with your local clinical team.

What will happen to the samples I give?

With your permission, the research blood samples provided will be stored at University of Birmingham in the UK. The samples will not be analysed as part of this study but will be used for future ethically approved medical/health research studies. Priority will be given to research related to IIH before being made available to other researchers. The samples will be labelled in a 'pseudo-anonymised' format where your data is anonymised to the people who receive and hold the samples but it contains information that can be used to link individual samples to clinical data for future studies. Any personal data will not be shared outside of the authorised members of the research team and the links will be kept secure in the Trials office – please see section 10 for full details.

All researchers requesting samples must have their research approved by a Research Ethics Committee. No access to blood samples will be allowed without permission from these committees.

Research may be carried out locally, in the UK or abroad, by academic, hospital or commercial organisations.

Samples may also be used to invent new treatments or tests for IIH, which could be patented. However, you will not be able to receive any money from these discoveries.

What happens when the trial stops?

You will return to regular clinical care. Your clinical team will continue to keep you informed about all treatments (standard of care or experimental) from clinic.

Will anybody get paid if I take part?

You will be able to claim some travel costs associated with some of your trial visits to hospital (in the first 12 months post-intervention). These payments are a maximum of £30 per visit for a total of 7 visits (Visits 3, 4, 5, 6, 7, 8, 9) if you provide receipts. Travel by car will be reimbursed at the standard NHS rate of your local Trust (typically 56p per mile). Your doctor will not be paid for including you in this trial. It is possible that you will need to attend additional appointments as a result of taking part in the trial. If you have difficulty in funding these costs, there are several ways in which your hospital might be able to help with expenses and/or travel for your treatment. You may be able to claim travel expenses using the Healthcare Travel Cost Scheme ran by the NHS. The link and information regarding this scheme can be found here:
https://www.nhs.uk/nhs-services/help-with-health-costs/healthcare-travel-costs-scheme-htcs/
Please speak to your Doctor if you would like further information.

What if relevant new information becomes available?

Sometimes the research team will receive new information about the treatment being studied. If this happens, your trial doctor will inform you and discuss with you whether you should continue in the trial. If you decide not to carry on, your trial doctor will make arrangements for your normal care to continue. If you decide to continue in the trial, he/she may ask you to sign an updated consent form. It is possible that your trial doctor might suggest you withdraw from the trial. He/she will explain the reasons and arrange for your care to continue. If the trial is stopped for any other reason, your local clinical team will tell you and arrange your continuing care.

What will happen if I don't want to carry on with the trial?

If you do not wish to carry on with the trial before the trial intervention, you will be offered the local NHS standard of care procedure followed by NHS follow up visits and no data will be collected after your withdrawal.

If you do not wish to carry on with the trial after you had the trial intervention, you will be followed up as per NHS routine follow-up. We would still like to collect information about your medical condition and treatment you may receive, including NHS Digital data, as this will be valuable to our research. If you have any objection to this please let your doctor know when you decide to withdraw from the trial. Data already collected prior to withdrawal will be kept and analysed.

Any stored blood samples that can still be identified as yours will be destroyed, if you wish.

Any data collected as part of this trial prior to your withdrawal of consent will be retained. This data is recorded in specific ways for the research to be reliable. This means that we won't be able to let you see or change the data we hold about you.

What if there is a problem?

If you have a concern about any aspect of this trial, you should ask to speak with the trial doctor who will do their best to answer your questions (see contact number at end of form). If you remain unhappy and wish to complain formally, you can do this through your hospital's Patient Advice and Liaison Services (PALS). Details can be obtained through your local clinical team.

In the event that something does go wrong and you are harmed during the trial then you may have grounds for legal action but you may have to pay your legal costs. The University of Birmingham has in place Clinical

Trials indemnity coverage for this trial which provides cover to the University for harm which comes about through the University's, or its staff's, negligence in relation to the design or management of the trial and may alternatively, and at the University's discretion provide cover for non-negligent harm to participants.

With respect to the conduct of the trial at Site and other clinical care of the patient, responsibility remains with the NHS organisation responsible for the clinical site and is therefore indemnified through NHS Resolution.

The University of Birmingham is independent of any pharmaceutical company, and as such it is not covered by the Association of the British Pharmaceutical Industry (ABPI) guidelines for participant compensation. The NHS have a duty of care to participants whether or not the participant is taking part in a clinical trial and the normal NHS complaints mechanisms will still be available to you.

What will happen to the results of the trial?

At the end of the trial, the information collected will be analysed and published in recognised medical journals. The identity of the participants who took part in the trial will remain confidential. The research team will also write a summary of the results with the patients' charity IIHUK to disseminate study results to the members of the public.
Should you wish to discuss the results of the trial, you should contact your trial Doctor; you will have the opportunity if you wish to be informed of the results of the trial once fully analysed.

Who is organising and funding the trial?

This trial is being sponsored by the University of Birmingham. It is funded by the National Institute for Health Research (grant number: NIHR131211).

How have patients and the public been involved in this trial?

Potential participants were involved in reviewing the Participant Information Sheet.

In designing this trial we have taken into account patient opinions on the frequency of participant visits and the tests that will be carried out.

Who has reviewed this Clinical Trial?

All research in the NHS is looked at by an independent group of people called a Research Ethics Committee, to protect your safety, rights, wellbeing and dignity. This trial has been reviewed and given favourable opinion by the West Midlands - South Birmingham Research Ethics Committee and by the NHS Health Research Authority (HRA). While the trial is ongoing, the results will be reviewed by an independent Data Monitoring Committee (DMC) and a Trial Steering Committee (TSC) to ensure that it is appropriate to continue with the trial.

10. CONFIDENTIALITY

Will the information I provide be treated as confidential?

Yes. All information collected about you for this trial will be subject to the General Data Protection Regulation (GDPR) and the Data Protection Act 2018 and will be kept strictly confidential. University of Birmingham is the Sponsor for this trial based in the UK. We will be using information from you, your medical records, your GP and NHS Digital for this research project and will act as the data controller for this trial. This means that we are responsible for looking after your information and using it properly. The University of Birmingham and the NHS will keep identifiable information about you for a maximum of 15 years after the trial has finished, allowing the results of the trial to be verified if needed.

All information collected by the Sponsor will be securely stored at the Cancer Research UK Clinical Trials Unit at the University of Birmingham (the Trials Office) on paper and electronically and will only be accessible by authorised personnel. The only people in the University of Birmingham who will have access to information that identifies you will be people who manage the trial or audit the data collection process. With your permission, your trial doctor will notify your GP that you are participating in the trial. They will also send a copy of your signed consent form in the post to the Trials Office to ensure that the correct consenting procedure has been carried out. This will have your name and signature on it.

The NHS will use your name and contact details to contact you about the research trial/send documents to you, and make sure that relevant information about the trial is recorded for your care, and to oversee the quality of the trial.

Under no circumstances will you be identified in any way in any report, presentation or publication arising from the data collected.

How will we use information about you?

We will need to use information from you, your medical records, your GP and NHS Digital for this research project.

This information will include:

- your name
- initials
- date of birth
- NHS number (if you have one or obtain it while participating to the trial)
- the first part of your postcode (before the space)

People will use this information to do the research or to check your records to make sure that the research is being done properly.

People who do not need to know who you are will not be able to see your name or contact details. Your data will have a code number instead (trial number or screening number).

We will keep all information about you safe and secure. Once we have finished the trial, we will keep some of the data so we can check the results. We will write our reports in a way that no-one can work out that you took part in the trial.

What are your choices about how your information is used?

You can stop being a part of the study at any time, without giving reason, but we will keep information about you that we already have.

If you choose to stop taking part in the study, we would like to continue collecting information about your health from your hospital and NHS Digital. If you do not want this to happen, tell us and we will stop.

We need to manage your records in specific ways for the research to be reliable. This means that we won’t be able to let you see or change the data we hold about you.

Where can you find out more about how your information is used?

You can find out more about how your information is used by:

- at www.hra.nhs.uk/information-about-patients/
- Accessing the CRCTU Privacy Policy available on our website: www.birmingham.ac.uk/research/crctu/data-protection.aspx
- Contacting the University's Data Protection Officer by email: dataprotection@contacts.bham.ac.uk

What data are we processing and for what purpose will we use it?

We will collect and process your personal data to conduct the research project, as explained in the Participation Information Sheet.

In addition to the data we collect directly from you, we will also collect health data relating to you from NHS Digital, such as hospital admissions and diagnostic imaging, which is routinely collected by NHS Digital.

What is our legal basis for processing your data?

The legal justification we have under data protection law for processing your personal data is that the trial is being performed in the public interest.

Who will my personal data be shared with?

Sometimes, external organisations assist us with processing your information, for example, in providing IT support. These organisations act on our behalf in accordance with our instructions and do not process your data for any purpose over and above what we have asked them to do. We make sure we have appropriate contracts in place with them to protect and safeguard your data. If your personal data are transferred outside the European Union (for example, if one of our partners is based outside the EU or we use a cloud-based app with servers based outside the EU), we make sure that appropriate safeguards are in place to ensure the confidentiality and security of your personal data.

By taking part in the trial you will be agreeing to allow research staff from the Trials Office to look at the trial records, including your medical records. It may be necessary to allow authorised personnel from government regulatory agencies (e.g. Medicines and Healthcare products Regulatory Agency (MHRA)), the Sponsor and/or NHS bodies to have access to information about you. This is to ensure that the trial is being conducted to the highest possible standards.

Data collected during the trial (i.e. pseudo-anonymised clinical data- data that can be directly linked to the clinical trial dataset) may be transferred, for the purpose of processing and analysing, to associated researchers and independent specialist reviewers within the European Economic Area and internationally (provided they agree to abide by UK General Data Protection Regulation). These details will be kept to the minimum required to perform the processing/analysis and will remain confidential within the trial research team.

Brain Images (MRI and/or CT scans), visual tests results and images and brain pressure recording (only for participants with VP shunt who have the pressure device) will be stored at University of Birmingham and/ or University Hospitals Birmingham, NHS Foundation Trust and then reviewed by experts in the UK or internationally. All identifiable information will be removed before transfer from the acquiring site. Your images will only be identified via your trial number (or screening number) and the date of acquisition. Expert doctors will have access to your pseudo-anonymised data to perform their review.

We will also need to record your NHS number to allow us to obtain your health data, such as hospital admissions and diagnostic imaging which is routinely collected by NHS Digital (for NHS sites in England only). Your NHS number and date of birth will be provided to NHS Digital for linkage purposes to allow us to obtain health data collected for up to 10 years after your trial intervention.

Additionally, when you agree to take part in a research study, the information about your health and care may be provided to researchers running other research studies in the University of Birmingham and in other organisations. These organisations may be universities, NHS organisations or companies involved in health and care research in this country or abroad. Your information will only be used by organisations and researchers to conduct research in accordance with the UK Policy Framework for Health and Social Care Research.

Your information could be used for research in any aspect of health or care, and could be combined with information about you from other sources held by researchers, the NHS or government.

Where this information could identify you, the information will be held securely with strict arrangements about who can access the information. The information will only be used for the purpose of health and care research, or to contact you about future opportunities to participate in research. It will not be used to make decisions about future services available to you, such as insurance or direct marketing.

Where there is a risk that you can be identified your data will only be used in research that has been independently reviewed by an ethics committee.

Informing General Practitioner / other healthcare practitioner

With your permission, your GP will be notified that you are taking part in this trial. Your GP will be sent a copy of this information sheet and a copy of your signed consent form. This communication is intended to ensure that your GP is informed of any treatment that you receive – so he/she can include this information in your GP medical records.

11. FURTHER INFORMATION AND CONTACT DETAILS

If you have any questions or concerns about your disease or this clinical trial, please discuss them with your doctor or nurse. We will provide you with a card indicating that you participate in this trial, the type of intervention you had and the contact details of a member of the team. You can get in touch with the doctors and nurses to discuss any doubts or worries you may have about the trial. Contact details are also shown below:

Trial Doctor Dr (insert name) Tel: (insert number)

Research Nurse(s) (insert name) Tel: (insert number)

Hospital Details (insert name and address of hospital)

Thank you for taking the time to read this information

Appendix 4: Exemplar IIH Intervention trial informed consent form

Informed Consent Form

IIH Intervention: A clinical trial comparing 2 treatments (shunts and stents) to preserve vision for people with Idiopathic Intracranial Hypertension (IIH)

**Site:**	....	**Participant Trial Number:**	☐☐☐
**Principal Investigator:**	....	**Trial Reference Number:**	RG_21-078

Please initial each box

1. I confirm that I have read and understand the Participant Information Sheet (version .... dated..........................) for the above trial. I have had the opportunity to consider the information, ask questions and have had these answered satisfactorily. ☐
2. I understand that my participation is voluntary and that I am free to withdraw at any time without giving any reason, without my medical care or legal rights being affected. ☐
3. I give permission for my date of birth and the first part of my postcode (before the space) to be given to the IIH Intervention Trials Office when I am registered to the trial as well as a copy of this consent form which will contain my name. ☐
4. I give permission for the collection and secure sharing of my NHS number and date of birth for data linkage purposes with NHS Bodies such as NHS Digital, and other similar regulatory authorities in order to extract and provide digital health data relevant to this study to the trial research team. ☐
5. I understand that the Trials Office will keep identifiable information about myself for a maximum of 15 years after the study has finished, to allow the results of the study to be verified. ☐
6. I understand that relevant sections of my medical notes and data collected during the trial may be looked at by individuals from the Trials Office, regulatory authorities, Sponsors and/or NHS bodies, where it is relevant to my taking part in this research. I give permission for these individuals to have access to my records. ☐
7. I understand that anonymised or pseudo-anonymised data collected during the trial (visual tests results and images, brain imaging and intracranial pressure recordings, if I have a ventriculoperitoneal shunt with a pressure monitoring device) will be transferred to the University of Birmingham and/ or the lead site, University Hospitals Birmingham NHS Foundation Trust, for this and future research analysis. ☐

8. I understand that anonymised or pseudo-anonymised data collected during the trial may be provided to other third parties (e.g. pharmaceutical companies or other academic institutions) for this and future research, safety monitoring or licensing purposes and will never be shared for purposes of insurance or marketing. This data may be provided to countries outside of the UK/European Economic Area. ☐

9. I agree to my GP being informed of my participation in this trial and to my GP being sent a copy of this consent form. ☐

10. I agree to take part in the above trial. ☐

The following are optional and will not affect entry into the trial, please initial in either the Yes or No boxes next to the following statements:

	**No**	**Yes**
OPTIONAL – I would like to be sent health-related questionnaires by email ahead of the trial visits when possible and agree to provide my email address to the hospital clinical team for this purpose only.	☐	☐
OPTIONAL – I agree that pseudo-anonymised blood samples are collected to be stored and used for future ethically approved medical/health research about IIH. I understand that future medical/health research may be carried in the UK or abroad, by academic, hospital or commercial organisations. I understand that I will not gain financially from any commercial involvement.	☐	☐

**Name of patient**	**Date**	**Signature**

**Name of person taking consent**<br>You must have signed the<br>Site Signature & Delegation Log	**Date**	**Signature**

If patient is unable to read the form, witness to provide full name, date and sign the form:

**Name of witness**	**Date**	**Signature**

Appendix 5: Additional secondary outcomes and exploratory outcome measures for IIH Intervention

Additional secondary outcome measures

Other vision outcome measures

- Visual acuity, measured by logarithm of the minimum angle or resolution (LogMAR) units over 6, 12 and 24 months
- Humphrey Visual Field (HVF) perimetric mean deviation (PMD) over 6, 12 and 24 months

Intervention reporting

- Proportion of participants re-presenting to hospital within 30 days
- Proportion of participants being re-admitted to hospital within 30 days

Outcomes to be recorded over 6, 12 and 24 months:

- Proportion of major complication, defined as the incidence of complications graded between III and V or major anaesthesia problems (including complications from revision procedures) according to the Clavien-Dindo classification of surgical complications [1]
- Proportion with minor complications, defined as Clavien-Dindo grade I-II [1] during hospital stay.
- Proportion of participants requiring revision in whom:
    - The revision intervention is the same as the primary intervention
    - The revision intervention crosses over to the alternative intervention
- Time to first revision
- Number of revisions per participant
- Proportion of participants with adverse events grade 3 or above as measured by the CTCAE version 5 [2]

Headache outcomes to be recorded over 6, 12 and 24 months

- Monthly headache days
- Monthly moderate to severe headache days
- Headache severity (numeric rating scale (NRS) 0-10)
- Moderate to severe headache severity (NRS $\geq$ 4)
- Monthly use of acute headache analgesics

Patient Reported Outcome Measures (PROM)

- Quality of Recovery 15 (QoR-15) measured at time of discharge [3]
- Satisfaction with intervention (5-point Likert scale) measured at time of discharge

Other outcomes to be recorded over 6, 12 and 24 months:

- Visual Function Questionnaire (NEI-VFQ-25 with 10-Item Neuro-Ophthalmic Supplement) [4, 5]
- Headache Impact Test (HIT-6) [6]
- 36-item Short form health survey (SF-36v2) [7]
- EuroQol 5 dimension 5 level survey (EQ5D-5L) [8]
- Time to return to work (if working)
- Work Productivity and Activity Impairment Questionnaire – General Health (WPAI–GH) [9]
- Proportion of participants requiring ICP lowering medication above a clinically relevant threshold

Healthcare resource use questionnaire

Exploratory Outcome Measures

Intervention / post-intervention:

- Dural venous sinus stenting (DVSS):
    - Pressure gradient across target stenosis pre-intervention
    - Pressure gradient across target stenosis immediately post-intervention
    - Proportion achieving technical success
    - Of the proportion not achieving technical success
        - Proportion where there is failure to deploy stent
        - Proportion where there is incorrect stent placement with respect to target stenosis(es)
        - Proportion where there is failure to reduce the stenosis after stent deployment
    - Characterisation of medical devices used within procedure
    - Characterisation of pre-procedure and post procedure imaging

- Cerebrospinal fluid (CSF) shunting:
    - Proportion achieving technical success
    - Of the proportion not achieving technical success
        - Proportion where there is incorrect placement of the proximal catheter (ventricles in VP shunt or subarachnoid space in lumboperitoneal shunt)
        - Proportion where there is incorrect placement of distal end of shunt (e.g. extraperitoneal)
        - Proportion with immediate shunt disconnection or kink on post-operative imaging
    - Characterisation of medical devices used within procedure
    - Characterisation of pre-procedure and post procedure imaging
    - Characterisation of Intracranial pressure

Characterisation of intervention first failure

- DVSS:

- Reason for DVSS failure as determined by site:
    - Proportion of participants with recurrence of original stenosis
        - Proportion in which original stenosis is associated with visual decline and/or recurrence of papilloedema
        - Proportion in which original stenosis is radiologically present in the absence of visual decline or recurrence of papilloedema
    - Proportion of participants with a new stenosis adjacent to stent
        - Proportion in which this stenosis is associated with visual decline and/or recurrence of papilloedema
        - Proportion in which this stenosis is radiologically present in the absence of visual decline or recurrence of papilloedema
    - Proportion of participants with a new stenosis not adjacent to stent
        - Proportion in which this stenosis is associated with visual decline and/or recurrence of papilloedema
        - Proportion in which this stenosis is radiologically present in the absence of visual decline or recurrence of papilloedema
    - Proportion of participants with stent thrombosis

- CSF shunting surgery:
    - Reason for CSF shunt failure as determined by site.
        - Proportion where there is incorrect placement of the proximal catheter (ventricles in ventriculoperitoneal shunt or subarachnoid space in lumboperitoneal shunt)
            - Proportion occurring in association with visual decline and/or recurrence of papilloedema
            - Proportion occurring in the absence of visual decline or recurrence of papilloedema
        - Proportion where there is incorrect placement of distal end of shunt (e.g. extraperitoneal)
            - Proportion occurring in association with visual decline and/or recurrence of papilloedema
            - Proportion occurring in the absence of visual decline or recurrence of papilloedema
        - Proportion with immediate shunt disconnection or kink on post-operative imaging
            - Proportion occurring in association with visual decline and/or recurrence of papilloedema
            - Proportion occurring in the absence of visual decline or recurrence of papilloedema
        - Proportion of participants with shunt infections
            - Proportion occurring in association with visual decline and/or recurrence of papilloedema
            - Proportion occurring in the absence of visual decline or recurrence of papilloedema

Other

- Body weight and body mass index (BMI) over 6, 12 and 24 months by trial arm
- Tinnitus Handicap Inventory (THI) [10] (measured over 6, 12 and 24 months) by trial arm

References

1. Dindo D, Demartines N, Clavien PA. Classification of surgical complications: a new proposal with evaluation in a cohort of 6336 patients and results of a survey. *Ann Surg*. 2004;240(2):205-213. DOI: 10.1097/01.sla.0000133083.54934.ae

2. Common Terminology Criteria for Adverse Events (CTCAE), Version 5.0 2017. Available at: https://ctep.cancer.gov/protocoldevelopment/electronic_applications/ctc.htm#ctc_50. Accessed 02-Dec-2024.

3. Stark PA, Myles PS, Burke JA. Development and psychometric evaluation of a postoperative quality of recovery score: the QoR-15. *Anesthesiology*. 2013;118(6):1332-1340. DOI: 10.1097/ALN.0b013e318289b84b

4. Mangione CM, Lee PP, Gutierrez PR, et al. Development of the 25-item National Eye Institute Visual Function Questionnaire. *Arch Ophthalmol*. 2001;119(7):1050-1058. DOI: 10.1001/archopht.119.7.1050

5. Raphael BA, Galetta KM, Jacobs DA, et al. Validation and test characteristics of a 10-item neuro-ophthalmic supplement to the NEI-VFQ-25. *Am J Ophthalmol*. 2006;142(6):1026-1035. DOI: 10.1016/j.ajo.2006.06.060

6. Kosinski M, Bayliss MS, Bjorner JB, et al. A six-item short-form survey for measuring headache impact: the HIT-6. *Qual Life Res*. 2003;12(8):963-974. DOI: 10.1023/a:1026119331193

7. Ware JE, Jr., Sherbourne CD. The MOS 36-item short-form health survey (SF-36). I. Conceptual framework and item selection. *Med Care*. 1992;30(6):473-483. DOI:

8. Herdman M, Gudex C, Lloyd A, et al. Development and preliminary testing of the new five-level version of EQ-5D (EQ-5D-5L). *Qual Life Res*. 2011;20(10):1727-1736. DOI: 10.1007/s11136-011-9903-x

9. Reilly MC, Zbrozek AS, Dukes EM. The validity and reproducibility of a work productivity and activity impairment instrument. *Pharmacoeconomics*. 1993;4(5):353-365. DOI: 10.2165/00019053-199304050-00006

10. Newman CW, Jacobson GP, Spitzer JB. Development of the Tinnitus Handicap Inventory. *Arch Otolaryngol Head Neck Surg*. 1996;122(2):143-148. DOI: 10.1001/archotol.1996.01890140029007

Appendix 6: Definitions of adverse events

Adverse event

Any untoward medical occurrence in a participant.

Serious adverse event

Any untoward medical occurrence or effect that:

- Results in death
- Is life-threatening*
- Requires hospitalisation** or prolongation of existing inpatients' hospitalisation
- Results in persistent or significant disability or incapacity
- Is a congenital anomaly/birth defect
- Or is otherwise considered medically significant by the Investigator***

Comments:

The term severe is often used to describe the intensity (severity) of a specific event. This is not the same as serious, which is based on patients/event outcome or action criteria.

* Life threatening in the definition of a serious adverse event (SAE) refers to an event in which the patient was at risk of death at the time of the event; it does not refer to an event that hypothetically might have caused death if it were more severe.

**Hospitalisation is defined as an unplanned, formal inpatient admission, even if the hospitalisation is a precautionary measure for continued observation. Thus, hospitalisation for protocol treatment, elective procedures (unless brought forward because of worsening symptoms) or for social reasons (e.g. respite care) are not regarded as an SAE.

*** Medical judgment should be exercised in deciding whether an adverse event (AE) is serious in other situations. Important AEs that are not immediately life threatening or do not result in death or hospitalisation but may jeopardise the participant or may require intervention to prevent one of the other outcomes listed in the definition above, should be considered serious.

Device deficiency

Inadequacy of a medical device related to its identity, quality, durability, reliability, safety or performance. This may include malfunctions, or inadequacy in the information supplied by the manufacturer.

Use error

Act or omission of an act that results in a different medical device response than intended by the manufacturer or expected by the user. Use error includes slips, lapses and mistakes. An unexpected physiological response of the participant does not itself constitute a use error.

Severity definitions

The following definitions will be used to determine the severity rating for all adverse events:

- **Mild**: Awareness of signs or symptoms that do not interfere with the participant's usual activity or are transient and resolved without treatment and with no sequelae.
- **Moderate**: A sign or symptom, which interferes with the participant's usual activity.
- **Severe**: Incapacity with inability to do work or perform usual activities.

Appendix 7: Expected serious adverse events

Table A7.1: Known post-CSF shunt complications (either lumbo- or ventriculo-peritoneal)

System Organ Class	Frequency of adverse events			
	**Very common**	**Common**	**Uncommon**	**Rare**
Infections and infestations		Shunt infection,<br>Meningitis,<br>Superficial surgical site infection	Brain abscess, Meningo-encephalitis, Ventriculitis<br>Epidural empyema<br>Peritonitis	
Immune system disorders				Anaphylaxis: contrast agent. Latex, device etc
Nervous system disorders	Headache	Seizure,<br>Brain haemorrhage (radiological),<br>Sciatica	Tonsillar herniation<br>Brain haemorrhage (clinical)/ stroke<br>Peripheral nerve/ brachial plexus injury<br>Radicular weakness (foot drop)	Cauda equina syndrome,<br>Spinal cord injury
Eye disorders		Visual symptoms		
Respiratory, thoracic and mediastinal disorders	Chest pain	Chest infection	Pneumothorax,<br>Lung injury<br>Anaesthesia related respiratory syndrome	Pulmonary embolus
Gastrointestinal disorders	Abdominal pain	Intra-abdominal pseudocyst formation	Bowel perforation,<br>Peritonitis	Liver injury,<br>Spleen injury,

System Organ Class	Frequency of adverse events			
	Very common	Common	Uncommon	Rare
		Abdominal infection		Abdominal vascular injury
Ear and labyrinth disorders	Transient hearing symptoms	Tinnitus,<br>Vertigo		
Renal and urinary disorders		Urinary tract infection		Kidney injury
Musculoskeletal system	Neck/ spinal (lower back)/ joint pain			Long bone/ joint/ head injury (intra-operatively)
General disorders and administration site conditions	Mechanical complications of shunt:<br>Shunt malposition, shunt blockage, shunt malfunction, shunt failure<br>Over drainage<br>Under drainage<br>Disconnection or fracture,<br>Kink<br>Withdrawal/ Migration	Wound healing problem/ erosion,<br>Dehiscence,<br>CSF leak,<br>Skin breakdown with hardware exposure<br>"Shuntalgia"<br>Venous thrombosis<br>Thrombophlebiti s		

Frequencies are defined according to the following convention:

Very common (≥ 1/10); Common (≥ 1/100 to < 1/ 10); Uncommon (≥ 1/1,000 to <1/100); Rare (≥ 1/10,000 to <1/1,000); Very rare (<1/10,000), not known (cannot be estimated from available data).

Table A7.2: Known post-DVSS complications

System Organ Class	Frequency of adverse events				
	Very Common	Common	Uncommon	Rare	Very Rare
Infections and infestations				Implant infection	
Neoplasms benign and malignant (including cysts and polyps)					Radiation induced neoplasms
Blood and lymphatic system disorders		Anticoagulant / antiplatelet complications			
Immune system disorders				Anaphylaxis: contrast agent. Latex, device etc	
Nervous system disorders	Worse headache (<5 days)	Worse headache (> 5 days),<br>Subclinical subdural / subarachnoid blood	Intracranial haemorrhage,<br>Stroke (permanent disability)		
Eye disorders			Visual loss (stent thrombosis)		
Cardiac disorders			Dysrhythmia	Myocardial infarction	
Vascular disorders		In stent narrowing or thrombosis,<br>New stenosis, adjacent stenosis<br>Stent migration	Non stent thrombosis, dural venous sinus thrombosis, deep vein thrombosis		
Respiratory, thoracic and mediastinal disorders			Anaesthesia related respiratory syndrome	Pulmonary embolus	

System Organ Class	Frequency of adverse events				
	Very Common	Common	Uncommon	Rare	Very Rare
Gastrointestinal disorders			Antiplatelet related gastroduodenal inflammation		
Skin and subcutaneous tissue disorders				Excess radiation (skin damage, hair loss)	
Ear and labyrinth disorders			Tinnitus,<br>Vertigo		
Renal and urinary disorders			Contrast nephropathy		
General disorders and administration site conditions		Vascular access site haematoma	Vascular access site thrombosis		

Frequencies are defined according to the following convention:

Very common (≥ 1/10); Common (≥ 1/100 to < 1/10); Uncommon (≥ 1/1,000 to <1/100); Rare (≥ 1/10,000 to <1/1,000); Very rare (<1/10,000), not known (cannot be estimated from available data).
