# Supplementary material for: Cerebrospinal fluid shunting or dural venous sinus stenting to preserve vision in idiopathic intracranial hypertension (IIH Intervention): protocol for an open-label, multicentre, randomised controlled phase IIb trial
Source: BMJ Open. 2026 Aug 3;16(8):e111013. doi: 10.1136/bmjopen-2025-111013 (PMC13436069; doi:10.1136/bmjopen-2025-111013)
Supplement: Supplementary file 2 [file bmjopen-2025-111013.reviewer_comments.pdf]

PEER REVIEW HISTORY

BMJ Open publishes all reviews undertaken for accepted manuscripts. Reviewers are asked to complete a checklist review form and are provided with free text boxes to elaborate on their assessment. These free text comments are reproduced below.

ARTICLE DETAILS

Title (Provisional)

Cerebrospinal fluid shunting or dural venous sinus stenting to preserve vision in idiopathic intracranial hypertension (IIH Intervention): Protocol for an open-label, multicentre, randomised controlled phase IIb trial

Authors

Tsermoulas, Georgios; Mollan, Susan P; Homer, Victoria; Kristunas, Caroline; White, Phil; Wakerley, Benjamin R; Toma, Ahmed K; Robertson, Fergus; Berman, Gabriele; Ahmed, Fayyaz; Atan, Denize; Bandyopadhyay, Soham; Barton, Darren; Booth, Thomas; Bremner, Fion; Denton, Amanda; Downer, Jonathan; Edwards, Richard; Frew, Emma; Gew, Jessie Jie Yi; Hill, Lisa; Hughes, Tom; Lax, Sian; Macdonald, Jason; Maxwell, Gemma; McHugh, James; Shah, Pushkar; Taleti, Esteban; Tasker, Rachel; Thomas, Shery; Joannides, Alexis J; Sinclair, Alex

---

VERSION 1 - REVIEW

**Reviewer**	**1**
**Name**	**Lenck, Stephanie**
**Affiliation**	**Paris Brain Institute**
**Date**	**12-Feb-2026**
**COI**	**None**

This manuscript addresses a timely and clinically relevant topic: comparing venous sinus stenting (VSS) versus cerebrospinal fluid (CSF) shunting in patients with severe idiopathic intracranial hypertension (IIH).

The study is conducted in an experienced center, with a well-defined and homogeneous population (patients with grade ≥4 papilledema). The inclusion criteria are clear, and the outcomes too.

Overall, the study is well-constructed and provides clinically useful data.

I have on Major Methodological Concern: The CSF shunt group includes both lumbar-peritoneal (LP) and ventriculo-peritoneal (VP) shunts. While understandable, this grouping introduces significant heterogeneity: Revision rates and complication profiles are distinct. Why not doing a triple randomisation or choosing one shunting technique ? Pooling them

may dilute or exaggerate complications, potentially biasing comparisons in favor of VSS, especially if LP shunts are predominant. A three-arm randomization (VSS / VP / LP) would have been methodologically stronger but would require to increase the sample size.

Minor requests :

Consider adding a flow diagram showing screened patients, exclusions, lost to follow-up, cross-overs, and re-interventions to improve clarity.

Interim analysis and monitoring: Except if I missed it, the manuscript does not specify whether any interim analyses, stopping rules, or Data Safety

---

VERSION 1 - AUTHOR RESPONSE

Reviewer: 1
Dr. Stephanie Lenck, Paris Brain Institute, Hopitaux Universitaires Pitie Salpetriere-Charles Foix
Comments to the Author:
This manuscript addresses a timely and clinically relevant topic: comparing venous sinus stenting (VSS) versus cerebrospinal fluid (CSF) shunting in patients with severe idiopathic intracranial hypertension (IIH).
The study is conducted in an experienced center, with a well-defined and homogeneous population (patients with grade ≥4 papilledema). The inclusion criteria are clear, and the outcomes too.
Overall, the study is well-constructed and provides clinically useful data.
I have on Major Methodological Concern: The CSF shunt group includes both lumbar-peritoneal (LP) and ventriculo-peritoneal (VP) shunts. While understandable, this grouping introduces significant heterogeneity: Revision rates and complication profiles are distinct. Why not doing a triple randomisation or choosing one shunting technique ? Pooling them may dilute or exaggerate complications, potentially biasing comparisons in favor of VSS, especially if LP shunts are predominant. A three-arm randomization (VSS / VP / LP) would have been methodologically stronger but would require to increase the sample size.
We thank the reviewer for this thoughtful comment and agree that LP and VP shunts differ in certain technical aspects and may have differing complication profiles. Our decision to group LP and VP shunts within a single "CSF shunt" comparator arm was deliberate and based on both methodological and pragmatic considerations.

First, current clinical practice for treating patients eligible for this trial varies substantially across centres, with some institutions predominantly using LP shunts and others VP shunts. Mandating a single shunt type – or requiring centres to randomise across three discrete surgical procedures – would have required substantial deviation from established local practice. This would likely have limited site participation, impeded recruitment, and ultimately reduced the generalisability of trial findings.

Second, although there are differences between LP and VP shunts, both techniques represent accepted standard-of-care CSF diversion procedures and are used interchangeably in routine management of this condition. The primary objective of the trial is to compare dural venous sinus stenting (DVSS) with *standard CSF diversion as practiced in contemporary clinical settings*, rather than to compare DVSS with a single, prespecified shunt subtype. For this reason, preserving the heterogeneity inherent in real-world shunting practice reflects clinical reality and enhances external validity.

Finally, while we agree that a three-arm trial could in theory provide additional granularity, it would require a substantially increased sample size (to greater than 200 participants), combined with the need for centres to deliver all three procedures it was deemed that this was not feasible within the funding, timelines, and clinical infrastructure available. The current two-arm design represents the most rigorous and pragmatic approach able to answer the central comparative-effectiveness question for this population.

To mitigate the different safety profiles, the reporting of treatment and safety parameters will be split by LP vs VP shunt.

Minor requests :
Consider adding a flow diagram showing screened patients, exclusions, lost to follow-up, cross-overs, and re-interventions to improve clarity.

We thank the reviewer for their suggestion. When we come to present the trial results we will include such a flow diagram as per CONSORT guidelines. However, as the trial is still ongoing and we are here presenting the methods of the protocol, we do not feel it appropriate and as per SPIRIT guidelines, the figure we have included summarises the patient pathway.

Interim analysis and monitoring: Except if I missed it, the manuscript does not specify whether any interim analyses, stopping rules, or Data Safety

There are no stopping rules or formal interim analyses, owing to the longitudinal nature of the primary outcome and the small sample size, we did not feel that including a formal interim analysis would be able to impact the design or running of the trial. Instead, ongoing unblinded primary outcome and safety data are provided to the independent data monitoring committee (DMC). This is included in the last paragraph on page 10.

The DMC was established to oversee participant safety and trial integrity throughout the study. The DSMC comprises clinicians and statisticians with relevant expertise who were not otherwise involved in the conduct of the trial.

The DMC has access to unblinded interim data, including treatment allocation, efficacy outcomes, and adverse event profiles. This unblinded access enabled the committee to perform an objective assessment of the balance between risks and benefits across study arms while maintaining the blinding of the investigators and trial team.

Annually, and additionally if required, the DMC reviews accumulating safety and efficacy data. Based on these reviews, the committee is responsible for making recommendations to the trial sponsor regarding continuation, modification, or termination of the study.

Specifically, the DMC could recommend early termination of the trial in the event of: (1) evidence of harm, such as a significant excess of adverse events in one arm; (2) clear evidence of benefit, where continuing the trial would be unethical; or (3) futility, where interim analyses indicated that the trial was unlikely to meet its primary endpoint.

All DMC deliberations and access to unblinded data are conducted in strict confidence. The investigators, participants, and sponsor remained blinded to treatment allocation unless unblinding was deemed necessary following a formal DMC recommendation.

This process will ensure that participant safety is continuously monitored while preserving the scientific validity and integrity of the trial.

This was summarised within the Trial Organisation Structure section, first paragraph on page 13.
